# Origin and function of short-latency inputs to the neural substrates underlying the acoustic startle reflex

**DOI:** 10.3389/fnins.2014.00216

**Published:** 2014-07-25

**Authors:** Ricardo Gómez-Nieto, José de Anchieta C. Horta-Júnior, Orlando Castellano, Lymarie Millian-Morell, Maria E. Rubio, Dolores E. López

**Affiliations:** ^1^Neuroscience Institute of Castilla y León, University of SalamancaSalamanca, Spain; ^2^Department of Cell Biology and Pathology, University of SalamancaSalamanca, Spain; ^3^Institute of Biomedical Research of Salamanca (IBSAL), University of SalamancaSalamanca, Spain; ^4^Department of Anatomy, Biosciences Institute, São Paulo State University BotucatuSão Paulo, Brazil; ^5^Department of Otolaryngology, University of PittsburghPittsburgh, PA, USA

**Keywords:** alertness system, binaural summation, cochlear root neurons, extracellular recordings, neuronal tracers, pontine reticular formation, rat, vglut1-auditory nerve

## Abstract

The acoustic startle reflex (ASR) is a survival mechanism of alarm, which rapidly alerts the organism to a sudden loud auditory stimulus. In rats, the primary ASR circuit encompasses three serially connected structures: cochlear root neurons (CRNs), neurons in the caudal pontine reticular nucleus (PnC), and motoneurons in the medulla and spinal cord. It is well-established that both CRNs and PnC neurons receive short-latency auditory inputs to mediate the ASR. Here, we investigated the anatomical origin and functional role of these inputs using a multidisciplinary approach that combines morphological, electrophysiological and behavioral techniques. Anterograde tracer injections into the cochlea suggest that CRNs somata and dendrites receive inputs depending, respectively, on their basal or apical cochlear origin. Confocal colocalization experiments demonstrated that these cochlear inputs are immunopositive for the vesicular glutamate transporter 1 (VGLUT1). Using extracellular recordings *in vivo* followed by subsequent tracer injections, we investigated the response of PnC neurons after contra-, ipsi-, and bilateral acoustic stimulation and identified the source of their auditory afferents. Our results showed that the binaural firing rate of PnC neurons was higher than the monaural, exhibiting higher spike discharges with contralateral than ipsilateral acoustic stimulations. Our histological analysis confirmed the CRNs as the principal source of short-latency acoustic inputs, and indicated that other areas of the cochlear nucleus complex are not likely to innervate PnC. Behaviorally, we observed a strong reduction of ASR amplitude in monaural earplugged rats that corresponds with the binaural summation process shown in our electrophysiological findings. Our study contributes to understand better the role of neuronal mechanisms in auditory alerting behaviors and provides strong evidence that the CRNs-PnC pathway mediates fast neurotransmission and binaural summation of the ASR.

## Introduction

**Figure 1:**
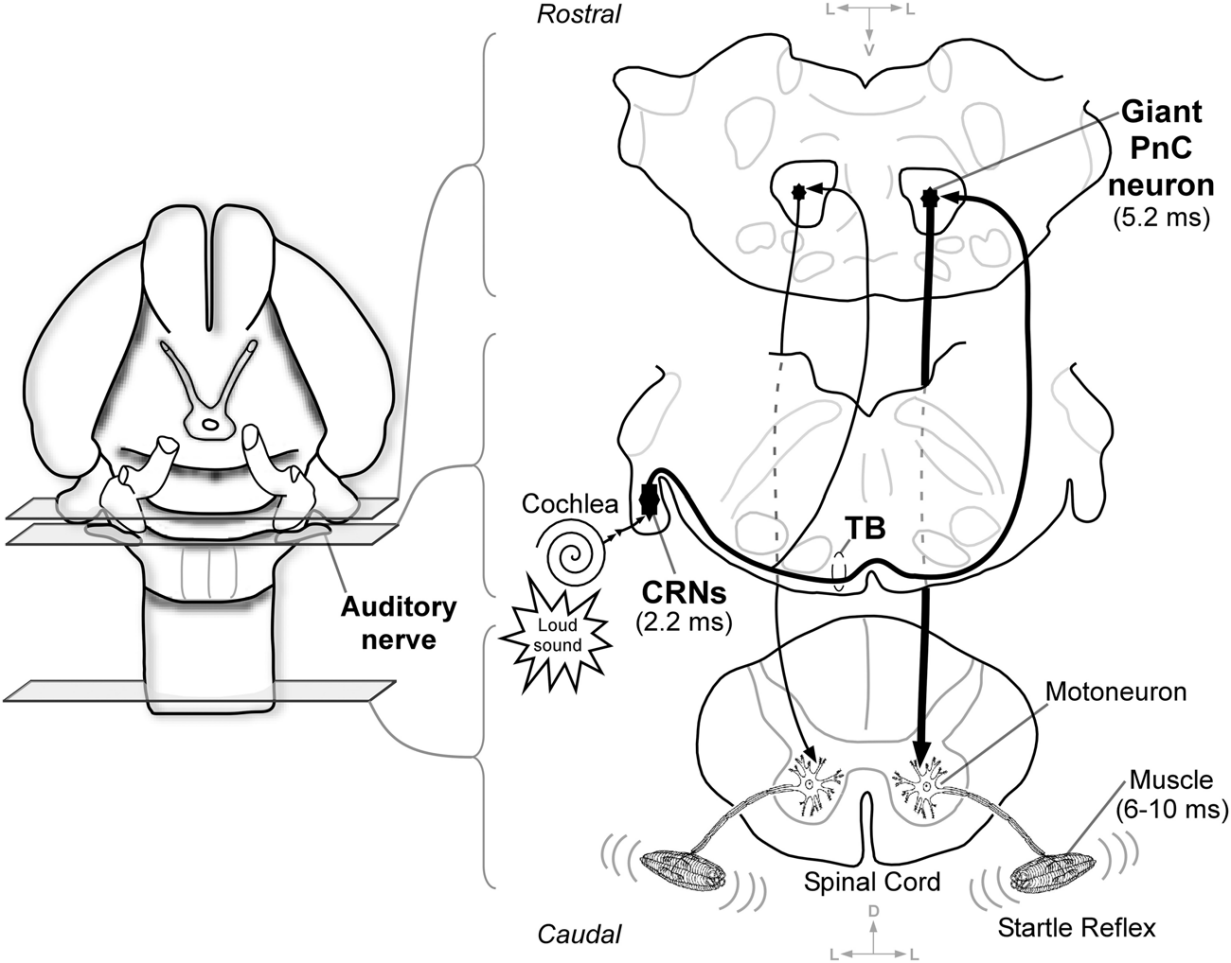
**Schematic drawing of the primary acoustic startle reflex in the rat brainstem based on the referred studies**. First, a sudden loud sound activates the auditory receptors in the cochlea. Next, auditory nerve fibers synapse cochlear root neurons (CRNs) which exhibit a secure response with first-spike latencies of approximately 2.2 ms (López et al., 1999; Sinex et al., 2001; Gómez-Nieto et al., 2010, 2013). This short-latency acoustic input is rapidly conducted to giant neurons in the pontine reticular formation (PnC) which respond with short-latencies of 5.2 ms (Lingenhöhl and Friauf, 1994; Lee et al., 1996; López et al., 1999). Finally, the acoustically driven PnC neurons send axons that contact motoneurons in the spinal cord to elicit an acoustic startle reflex with electromyographic latencies of 6–10 ms (Ison et al., 1973; Davis et al., 1982; Caeser et al., 1989). The line width represents side predominance within the circuit which is shown in coronal brainstem sections. Notice the axons of CRNs course through the trapezoid body (TB) to target preferentially contralateral PnC neurons (López et al., 1999; Nodal and López, 2003). Arrowheads indicate the flow of acoustic information within the circuit. Projections from CRNs to other non-auditory nuclei which participate in the full expression of the acoustic and pinna reflexes are not shown in this scheme (see details in López et al., 1999; Horta-Júnior et al., 2008).

The acoustic startle reflex (ASR) is a survival mechanism of alarm, which rapidly alerts and arouses organisms to a sudden loud auditory stimulus. Behaviorally, the ASR involves a rapid and sequential activation of muscles along the length of the body as well as an autonomic physiological response (Prosser and Hunter, 1936; Szabo, 1964; Hoffman and Ison, 1980). The ASR and its modulations, which are sensible to a variety of experimental approaches, can be easily tested in humans and rodents (Braff and Geyer, 1990; Lehmann et al., 1999). Thus, the ASR has been consolidated as an important research tool for studying brain mechanisms of learning, memory, emotions, sensory gating, and movement control (Davis and Sheard, 1974; Davis, 1990; Lang et al., 1990; Yeomans and Frankland, 1996; Koch, 1999; Swerdlow et al., 2001). The rat is an excellent animal model to study the ASR, and hence, the neuronal circuits underlying the ASR in rats are of great interest. It is well established that a relatively simple pathway in the brainstem mediates the ASR (Figure 1). The cochlear root neurons (CRNs), true sentinels of the rodent auditory pathway, are the first brainstem neurons receiving direct input from spiral ganglion cells (Harrison et al., 1962; Merchán et al., 1988; Osen et al., 1991; López et al., 1993, 1999). Collectively, they comprise the so-called cochlear root nucleus, and are morphologically characterized by their large cell body and thick dendrites that distribute among the eighth nerve fibers (Merchán et al., 1988; López et al., 1993). The thick myelinated axons of CRNs course through the trapezoid body (TB) to innervate neurons in the caudal pontine reticular nucleus (PnC) on both sides of the brainstem, with a clear contralateral predominance (López et al., 1999; Nodal and López, 2003). Finally, the acoustically driven PnC neurons project to facial, cranial and spinal motoneurons that rapidly activate the muscle contractions (Lingenhöhl and Friauf, 1994; Lee et al., 1996; Yeomans and Frankland, 1996; Koch and Schnitzler, 1997). The electromiographic latencies of the muscles during the ASR are extremely short (6–10 ms) (Ison et al., 1973; Davis et al., 1982; Caeser et al., 1989). Such short-latency motor response is consistent with the neuronal latencies observed in the primary ASR mediating circuit (Figure 1). Thus, CRNs exhibit a secure electrophysiological response to tone burst, with first-spike latencies of approximately 2.2 ms (Sinex et al., 2001; Gómez-Nieto et al., 2010, 2013), and giant PnC neurons that receive acoustic inputs show first-spike latencies of 5.2 ms (Lingenhöhl and Friauf, 1994). The main purpose of this study is to reappraise the anatomical origin of short-latency auditory inputs to CRNs and PnC neurons and investigate whether the CRNs-PnC neuronal pathway is responsible for the binaural summation that occurs during the ASR. Although much is currently known about the cochlear nerve projection to the cochlear root nucleus (Harrison et al., 1962; Merchán et al., 1988; Osen et al., 1991; López et al., 1993, 1999), the neuroanatomical and neurochemical mechanisms by which CRNs integrate fast and frequency-specific acoustic information has not been clearly established. The rats are extremely sensitive to high frequency sounds, and the amplitude of the ASR depends on sound features such as intensity, duration and frequency (Gourevitch and Hack, 1966; Błaszczyk and Tajchert, 1997). Since the characteristic frequency of CRNs is approximately 30 kHz (Sinex et al., 2001; Gómez-Nieto et al., 2013), we aimed to determine whether auditory nerve afferents tonotopically innervate CRNs. The primary auditory afferents use glutamate as neurotransmitter (Hackney et al., 1996; Furness and Lawton, 2003; Rubio and Juiz, 2004) and their endings contain the vesicular glutamate transporter 1 (VGLUT1) as it has shown in other areas of the cochlear nucleus complex (Zhou et al., 2007; Gómez-Nieto and Rubio, 2009). Here, we demonstrated that VGLUT1 is also associated with auditory nerve afferents that terminate onto CRNs, which suggests a fast mechanism of controlling and securing excitatory transmission in the cochlear root nucleus. Additionally, there are two questions that have been raised about the primary ASR circuit at the level of the PnC. One question is whether, in addition to the CRNs, other neuronal types within the cochlear nucleus complex provide short-latency acoustic inputs to PnC neurons, as proposed by other researchers (Davis et al., 1982; Kandler and Herbert, 1991; Lingenhöhl and Friauf, 1994; Meloni and Davis, 1998). The other question relates to the neuronal bases underlying the binaural summation of the startle reflex, which has yet to be elucidated (Marsh et al., 1973; Li and Frost, 1996; Yeomans et al., 2002). To address these issues, we used extracellular recordings *in vivo* followed by subsequent tracer injections to investigate the sound evoked responses of PnC neurons after contra-, ipsi-, and bilateral stimulation, and identify the source of their auditory afferents. Our electrophysiological study combined with neuronal tracing indicated the existence of a binaural summation process in the pontine reticular formation, and demonstrated that CRNs are the main source of short-latency acoustic inputs to PnC neurons. Additional anterogradely tracing experiments were also performed to verify whether neurons of the dorsal and ventral cochlear nucleus (DCN and VCN, respectively) innervate PnC neurons. Furthermore, we correlated our electrophysiological findings with changes in the ASR behavioral performance of monaural earplugged rats. Monaural earplugging reduces auditory nerve activity and affects processing through the auditory pathways (Potash and Kelly, 1980). Since the startle depends on normal binaural auditory interaction (reviewed in Yeomans et al., 2002), we found a reduction of the ASR amplitude in rats under monaural sound-deprivation conditions, which was consistent with the PnC neurons' response to monaural acoustic stimulation. Our study provides convergent anatomical, electrophysiological and behavioral evidence for the role of the CRNs-PnC projection in fast neurotransmission and binaural summation of the ASR and offers new insights into the structure and function of the primary ASR neuronal circuit.

## Materials and methods

### Experimental animals

In total, 36 adult female Wistar rats (Charles River Laboratories) weighing 165–320 g were used in this study. The animals were randomly housed and maintained under normal 12/12 h light/dark cycle (lights on at 07:00 h) in a temperature -and humidity-controlled environment. The rats were given *ad libitum* access to food and water over the study period. The experiments were conducted in compliance with the guidelines for the use and care of laboratory animals of the European Communities Council Directive (2010/63/EU), the current Spanish legislation (RD 1201/05), and with those established by the Institutional Bioethics Committee. All efforts were made to minimize the number of animals used. For the surgical procedures, the animals were deeply anesthetized with a mixture of ketamine (40 mg/kg body weight) and xylazine (7 mg/kg body weight). The suffering of the animals was minimized during the surgery by monitoring the depth of anesthesia carefully. Supplementary doses of anesthetic were administrated as required to maintain deep anesthesia throughout the duration of the experiment.

### Neuroanatomical experiments: surgery, tracer injections, and tissue processing

A total of 17 animals were used in the neuroanatomical experiments with the aim of studying the source of acoustic inputs to CRNs and PnC Neurons. To investigate the distribution of cochlear nerve inputs on CRNs, 4 rats received anterograde horseradish peroxidase (HRP, type VI, Sigma, St Louis, MO) injections into basal and apical regions of the cochlea following the procedure described by Osen et al. (1991). Once anesthetized, the tympanic bulla was exposed and a small orifice was made in the lateral wall of the cochlea at various apico-basal levels. HRP (20% in distilled water) was pressure-injected through a glass micropipette for 10–20 min, and a second micropipette was placed in the oval window for fluid removal. After the wound was washed and sutured, the animals were allowed to recover for 2 days. In cases with electrophysiological recordings in the right PnC (see below), we injected the bidirectional tracer, biotinylated dextran amine (BDA, 10,000 MW; #D-1956; Molecular Probes, Eugene, OR) to verify the recording site and investigate the source of cochlear nucleus inputs to PnC neurons. In another set of experiments, 6 rats received unilaterally injections of BDA into the left DCN and VCN. All surgical and stereotaxic procedures for injecting the BDA were identical to that used in our previous studies (Gómez-Nieto et al., 2008a, 2013; Horta-Júnior et al., 2008). BDA (10% in distilled water) were injected iontophoretically via a glass micropipette (25μm tip diameter), with 3μA positive current pulses (7 s on/7 s off) for a period of 15 min. The stereotaxic coordinates for the right PnC was based on previous reports which studied afferent projections from auditory brainstem nuclei into the PnC (Lingenhöhl and Friauf, 1994; Lee et al., 1996; López et al., 1999; Nodal and López, 2003). The coordinates for the left VCN were precisely the same as those used by (Gómez-Nieto et al., 2013). All sterotaxic coordinates, including those for the DCN, were obtained from the atlas of the rat brain (Paxinos and Watson, 2009), using an electrode angle calibrator (David Kopf Instruments). After the tracer injection, the scalp was sutured and the animal was allowed to recover for a minimum of 7 days. Tissue preparation for light microscopy included: perfusion of the animals, brain dissection and subsequent cryoprotection with sucrose, freezing and serially slicing at 40μm thickness along parasagittal and coronal planes, visualization of HRP and BDA neurotracers, and calbindin protein-D28K (CaBP) immunohistochemistry. All these techniques were applied in a manner identical to that used in our previous reports (Osen et al., 1991; López et al., 1993, 1999; Gómez-Nieto et al., 2008a, 2013). In cases with HRP injections, we followed a standard immunostaining protocol to visualize CaBP as described by Gómez-Nieto et al. (2008a). CaBP immunoreactivity has been extensively utilized for detecting the cell body and dendrites of the CRNs (López et al., 1993; Gómez-Nieto et al., 2008a, 2013). Thus, nickel-intensified peroxidase reaction was developed for HRP visualization in order to distinguish from CaBP immunohistochemistry without heavy metal intensification of diaminobenzidine (Gómez-Nieto et al., 2008a). Detailed information of the antibodies and dilutions used for CaBP immunohistochemistry is shown in Table 1. All sections processed for light microscopic analysis were mounted on slides and 4 alternate series were counterstained with cresyl violet to highlight cytoarchitectonic divisions; the other sections were dehydrated in ethanol and coverslipped with Entellan Neu (#107961; Merck, Darmstadt, Germany).

**Table 1:** **List of antibodies and dilutions used in the immunohistochemistry techniques**.

**Antigen**	**Primary antibody**^†^	Catalog/Brand^*^	**Dilution**	**Secondary antibody**	**Dilution**	Catalog/Brand^*^	**Processing^‡^**	**References**
*CaBP*	Mouse anti-CaBP	#C-8666/Sigma	1:200	Biotinylated horse anti-mouse	1:200	#BA-2000/Vector	LM	Gómez-Nieto et al., 2008a,b, 2013
*VGLUT1*	Rabbit anti-VGLUT1	#135-302/Synaptic Systems	1:1000	Cy3 goat anti-rabbit	1:200	#111-165-003/JI	CM	Gómez-Nieto et al., 2008b; Gómez-Nieto and Rubio, 2009
				Cy5 goat anti-rabbit	1:200	#115-175-003/JI		
*c-Fos*	Rabbit anti-c Fos	#SC-52/SCB	1:2500	Biotinylated goat anti-rabbit	1:200	#BA-1000/Vector	LM	Castellano et al., 2009, 2013

* *Antibody manufacturers: JI, Jackson Immunoresearch, West Grove, PA, USA; SCB, Santa Cruz Biotechnology (Santa Cruz, CA, USA); Synaptic Systems, Göttingen, Germany; Sigma, Sigma-Aldrich, St Louis, MO, USA; Vector, Vector Laboratories, Burlingame, CA, USA*.

† *Specificity and immunogen sequence of primary antibodies: (1) The anti-CaBP was a mouse IgG isotype produced by hybridization of mouse myeloma cells with spleen cells from mice immunized with calbindin D-28k purified from chicken gut. The antibody specifically stains the 45Ca-binding spot of the calbindin D-28k (MW 28 kDa) of tissue originating from rat brain in two-dimensional immunoblots; (2) The anti-VGLUT1 is a polyclonal antibody generated in rabbits against Strep-Tag fusion protein containing amino acid residues 456–560 of rat VGLUT1. This antibody has been tested in preadsorption experiments that blocked efficiently and specifically the corresponding signals (manufacturer's technical information; see also Zhou et al., 2007); (3) The anti-c Fos is a is a rabbit polyclonal antibody produced against a epitope mapping at the N-terminus of c-Fos of human origin (see details in manufacturer's technical information)*.

‡ *Tissue processed for light microscopy (LM) or confocal microscopy (CM)*.

#### Double fluorescent tract-tracing experiments

To label CRNs and the cochlear nerve, we used identical procedures to those used in our previous studies (Gómez-Nieto et al., 2008b; Gómez-Nieto and Rubio, 2009). Three animals received a unilateral injection of the fluorescent tracer, dextran fluorescein isothiocyanate (D-FITC; #D-1820; Molecular Probes, Eugene, OR), into the left TB to label CRNs retrogradely. The stereotaxic coordinates targeted the course of CRN axons which project to PnC neurons via the TB (López et al., 1999). A volume of 0.15–0.2μl of D-FITC (10% in distilled water) were pressure-delivered with a Hamilton syringe (#710; Hamilton Co., Reno, NV) attached to a Stereotaxic Injector (Stoelting Co., Wood Dale, IL). After the scalp was sutured, the animals were allowed to recover for 4–7 days. Then, the animals were deeply anesthetized and perfused through the heart with 0.5% paraformaldehyde (PFA) in 0.1 M phosphate buffer, pH 7.4. The cochlea was removed, and crystals of the lipophylic dye DiI (#D-3911; 1.1′-dioctadecyl-3,3,3′,3′-tetramethylindocarbocyanine perchlorate; Molecular Probes) were placed on the exposed auditory nerve. The brains were stored in 4% PFA for approximately 30 days at room temperature protected from light and were processed for confocal microscopy as described by Gómez-Nieto and Rubio (2009).

#### VGLUT1 immunofluorescence and colocalization experiments

Two rats were used to identify VGLUT1 immunopositive terminals in the cochlear root nucleus. The immunofluorescence was performed following the same protocol described by Gómez-Nieto and Rubio (2009). After obtaining the brain tissue, free-floating sections (40μm in thickness) were pretreated with phosphate buffer saline (PBS) 0.3% TritonX-100, and blocked for 1h at room temperature with 6% normal goat serum (#S-1000, Vector Laboratories) in PBS. Subsequently, the brain slices followed overnight incubation at 4°C with the primary antibody (see Table 1 for complete information of antibodies). Thereafter, the sections were rinsed extensively in PBS and incubated for 1 h at room temperature with their corresponding fluorophore conjugated secondary antibody (Table 1). In another set of experiments, we studied the codistribution of cochlear nerve terminals and VGLUT1 on CRNs retrogradely labeled with D-FITC. For this purpose, we analyzed 2 animals from our previous studies in which a triple-labeling procedure was designed to detect colocalization of VGLUT1 with auditory nerve terminals on bushy cells dendrites (Gómez-Nieto and Rubio, 2009). These animals received an injection of D-FITC in the TB and insertions of DiI crystals in the cochlear nerve root, and finally the brainstem sections were processed for VGLUT1 immunofluorescence as described above. In these colocalization experiments, we used Cy5 goat anti-rabbit (blue emission) to distinguish the signal from the DiI (red emission)-labeled auditory nerve endings. Finally, all sections were rinsed extensively in PBS, dipped briefly in distilled water, mounted onto fluorescence-free slides, air-dried, and coverslipped with ProLong Antifade Kit (#P7481; Molecular Probes) to prevent photobleaching. In all immunofluorescence experiments, negative controls were not treated with primary antibodies, and this resulted in the complete absence of immunolabeling.

### Electrophysiology experiments combined with BDA injections

Seven rats were used for extracellular multi-unit recordings of acoustically driven PnC neurons, and subsequent neuronal tract-tracing study. The animal's body temperature was monitored and maintained at 38°C by a thermostatically controlled electrical blanket. Once anesthetized, each animal was placed inside a sound-attenuated room in a stereotaxic frame in which the ear bars were replaced by a hollow speculum coupled to a sound delivery system (Sony MDR E-868 earphones). The output of the system at each ear was calibrated *in situ* using a DI-2200 spectrum analyzer and a Brüel and Kjær 4134 microphone. This standard calibration was used to set the levels of tones and white noise in all experiments. Both pure tones and bursts of white noise were generated using a waveform generator (Hewlett Packard-8904A multifunction synthesizer) controlled by a computer. Acoustic stimuli were delivered to the ears monaurally, ipsi- and contralateral to the recording site, as well as binaurally. After craniotomy and brain surface exposure, a glass micropipette with the tip broken to a diameter of 10μm was filled with 10% of BDA in 2M NaCl (15–25 MΩ) and lowered through the brainstem using a piezoelectric microdrive (Burleigh 6000 ULN, Burleigh Instruments, Fishers, NY) with a resolution of 1μm. The electrode was aimed to target PnC neurons in the right side of the brainstem following the same stereotaxic approach described above. As the micropipette was advanced into the brainstem, we used white noise as search stimuli. Extracellularly recorded action potentials were amplified (×10,000; BAK MDA-4I), filtered (0.3–3 kHz), discriminated (BAK DIS-I) and time-stamped with an accuracy of 10μs by a CED-1401plus Laboratory Interface (Cambridge Electronic Design). Once significant evoked activity was detected, bursts of white noise and pure tones were used as experimental stimuli to assess the modifications of PnC neural responses at different conditions (intensity, frequency and side of stimuli presentation). Stimuli were 100 presentations of 75 ms pure tones (5 ms rise/fall time) or noise bursts at a rate of 2/s. Sound level was varied from 60 to 90 dB in 10-dB steps and frequency from 0.5 to 35 kHz. These intensity and frequency ranges were chosen based on the high firing thresholds and broad frequency tuning of PnC neurons (Lingenhöhl and Friauf, 1994). The spikes times evoked by the different stimuli were stored and were used to calculate the response magnitude (spikes per ms) and first spike latencies. The integration window to calculate the mean firing rate was the first 25 ms after stimuli presentation. For each recording, we obtained peristimulus time histograms to display the responses to the stimuli. Data from first spike latencies and responses of PnC neurons were pooled for statistical comparison between stimulation with pure tones and noise bursts as well as the side of stimuli presentation. At the end of each experiment, BDA was injected iontophoretically following the same procedure described above. Finally, the animal was allowed to recover for a minimum of 7 days and followed the histological procedure to visualize the neuronal tracer at light microscopy (López et al., 1999; Gómez-Nieto et al., 2008a, 2013).

### Behavioral experiments

A total of 12 rats were used to study the behavioral paradigm of the ASR under monaural sound-deprivation conditions. The animals were randomly divided into one experimental group with monaural earplugging (*n* = 6) and an intact control group (*n* = 6). The ASR amplitude was assessed in rats from the experimental group prior to and following 4 days of monaural earplugging, and their ASR was compared with those from the control group. After the behavioral test, the animals were kept at rest during 60 min, and then followed the tissue preparation to detect c-Fos immunoreactivity at light microscopy (see below).

#### ASR apparatus (system) and procedure

Before testing, the rats were habituated to the experimental conditions, especially to their placement into the ASR apparatus. All testing was carried out between 9:00 A.M. and 11:00 A.M, using the SR-LAB system (SDI, San Diego, CA, USA) as described by Castellano et al. (2009). The acoustic startle reflexes were measured in six identical startle response systems (SR-LAB). Acoustic stimulus intensities and response sensitivities were calibrated (using an SR-LAB Startle Calibration System) to be nearly identical in each of the six SR-LAB systems (maximum variability <1% of stimulus range and <5% of response ranges). Each system consisted of a nonrestrictive Plexiglas cylinder, 8.7 cm in internal diameter and 20.5 cm long, mounted on a platform which was located in a ventilated, sound-attenuated chamber. Cylinder movements were detected by a piezoelectric accelerometer mounted under each platform and were digitized and stored by an interfacing computer assembly. A loudspeaker mounted 14.5 cm above the cylinder provided the broadband background noise and acoustic stimuli. Each testing session consisted of an acclimatization period of 5 min and 64 trials with a 30 s interval between them. The trials were a single noise pulse (20 ms bursts of white noise) presented at different intensities (95, 105, and 115 dB SPL) in a random manner. Whole body ballistic movements corresponding to startling responses were collected and analyzed by the SR-LAB system providing two main values of interest: *V*_max_ and *T*_max_. The *V*_max_ represents the peak startle response (ASR amplitude) that occurs during each trial while *T*_max_ is the time from stimulus to the peak startle response (ASR latency). The background noise of 65 dB SPL was generated throughout the entire session in order to avoid interference from external noise and ensure equal experimental conditions.

#### Monoaural earplugging

After ASR testing, animals from the experimental group were anesthetized and put on a warm blanket under a stereomicroscope. The skin was disinfected and foam earplugs (Moldex®, Culver City, CA) were cut to appropriate size and introduced into the right external canal as described by Whiting et al. (2009). Once the ear canal was sealed, the animal was maintained under normal conditions for 4 days until the ASR was measured again. After earplugging, the animals showed no symptoms of stress or infection, and kept their weight steady (see table in Supplemental Figure 6). Acoustic effects of monaural earplugging in rats include attenuation of approximately 40 dB on the same side to the earplug as well as modifications of the threshold, amplitude and latency of acoustic brain response waveforms (Whiting et al., 2009; Popescu and Polley, 2010; Wang et al., 2011).

#### Fos immunohistochemistry

Once the behavioral tests were completed, animals from the control and the monaural experimental group were processed to detect c-Fos immunoreactivity in the auditory pathway, and more specifically in the inferior colliculus (IC). The noise bursts used to measure the ASR served to induce the c-Fos expression in the auditory pathway. Since c-Fos protein has been widely used as a marker of early neuronal activation (Sagar et al., 1988; Dragunow and Faull, 1989; Murphy and Feldon, 2001), the quantification c-Fos immunoreactivity in the IC allowed us to check the efficiency of the earplugging. Perfusion of the animals, serial brain slicing into 40μm coronal sections and c-Fos immunohistochemical staining protocol was described in detail elsewhere (Castellano et al., 2009, 2013). Sections were incubated in primary antiserum against c-Fos protein for 72 h at 4°C. The tissue was then washed and incubated with its corresponding biotinylated secondary antibody for 2 h at room temperature, and finally visualized with the avidin-biotin-peroxidase complex procedure (Vectastain, Vector Labs.) and histochemistry for peroxidase without heavy-metal intensification. Complete information of the primary and secondary antibodies used for c-Fos immunohistochemistry is provided in Table 1. Immunolabeling was abolished by omission of the primary antibody. For each brain, all sections were mounted on slides, dehydrated and coverslipped as described above.

### Image, data, and statistical analysis

The sections processed for light microscopy were examined on an upright brightfield microscope (#BX5; Olympus, Center Valley, PA, USA) equipped with a digital camera (SpotRt®; Diagnostic Instruments, Sterling Heights, MI, USA). Low-magnification images were taken with the 4×, 10×, or 20× objective lens, and high magnification images were taken with a 40× or 100× objective lens (oil immersion) for morphometric measures of labeled structures. The morphometric analysis was carried out with ImageJ (version 1.42; Rasband W.S., ImageJ, Bethesda, Maryland, USA, U.S. National Institutes of Health; 1997–2011. http://imagej.nih.gov/ij/). In the behavioral experiments, we estimated the number of c-Fos-immunolabeled neurons in the IC using a similar quantitative analysis as previously described by Castellano et al. (2013). High magnification micrographs (×40) of the ipsilateral and contralateral IC were taken with a Leica microscope (model # DMRB) coupled to a motorized *x*–*y*–*z* motor stage, and connected to a PC with Stereo Investigator software (MicroBrightField, VT, USA). The stereological analysis was accomplished with the aid of the optical fractionator method (Gundersen et al., 1988). We selected one section containing the left and right IC in each one of the 12 animals used in the behavioral experiments. To guarantee the homogeneity and the reproducibility of the area of interest, the contour of the IC was drawn at low magnification (2x), using transparent templates taken from the atlas of the rat brain (Paxinos and Watson, 2009) at 0.2 mm anterior from the interaural plane. The templates were placed on the screen of the computer and served to select the same area of interest in all histological sections. After that, the Stereo Investigator system automatically estimated the size of the counting grid (optical dissector) and the number of zones to be sampled. The neurons were counted according to the optical dissector counting rules as they first came into focus from 3μm below the upper surface of the section and did not touch the right inferior and superior edges of the dissector. Quantification was carried out in a single-blind assessment by two different investigators and only neurons that displayed a clearly staining above the background were considered as a count. For the immunofluorescence and colocalization experiments, the sections were examined with a conventional brightfield microscope equipped with epifluorescence. In selected slides, the sections were analyzed with a Leica TCS SP2 confocal laser scanning microscope (Leica Microsystems, Mannheim, Germany) coupled to a Leica DM IRE2 inverted microscope and equipped with argon and helium neon lasers with excitation wavelengths of 458, 476, 488, 543, 568, and 633 nm. The fluorochromes FITC, Cy3, DiI, and Cy5 were detected sequentially, stack by stack, with the acousto-optical tunable filter system and triple dichroic mirror TD488/543/633. The background was controlled, and the photomultiplier voltage (800 V) was selected for maximal sensitivity in the linear range. The objectives used were oil immersion ×40 and ×63/numerical aperture 1.30, providing a resolution of ×150 nm in the xy plane and ×300 nm along the z-axis (pinhole 1 Airy unit), as well as several electronic zoom factors up to ×1.58. To determine the codistribution of the immunolabeled terminals, series of 25–50 confocal images were obtained to generate a maximal-intensity z projection of stacks and an orthogonal projection (= xy, xz, yz planes, for z stacks series). Colocalization of the fluorochromes within positive terminals was verified in the orthogonal view. All photomicrographs shown in the figures were processed with minor modifications in brightness, contrast and to remove the tissue free background using Adobe Photoshop® CS3 Extended (Version 10.0) and assembled in Canvas 7.0 software. Statistical analyses were performed using the SPSS software, version 18.0 (SPSS Inc., Chicago, IL, USA). All mean values were expressed ± the standard error of the mean. Comparisons between groups were made by analysis of variance (mixed ANOVA split-plot), with pairwise comparisons Sheffe (between-subjects analysis) and Bonferroni post-hoc test (intra-subject analysis). To compare differences between two means, we used Student's t-test taking into account the Levene's test for equality of variances. The differences between groups were regarded as statistically significant when *p* ≤ 0.05.

## Results

### HRP injections into the basal and apical coils of the cochlea

**Figure 2:**
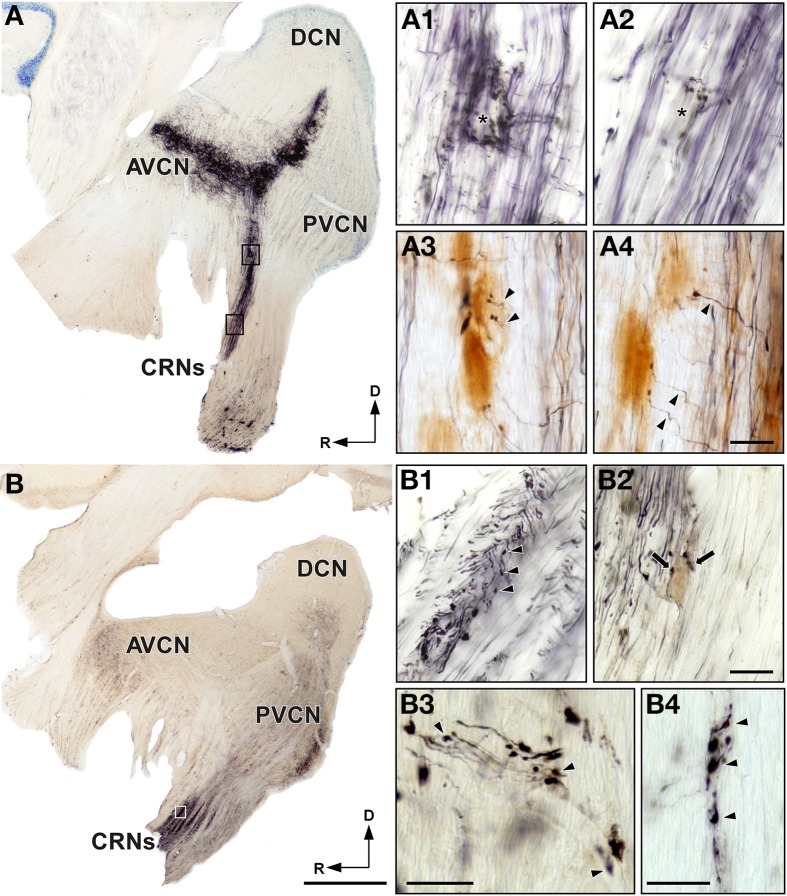
**Injections of HRP in the basal and apical regions of the spiral ganglion generate respectively labeled auditory nerve endings on the cell body and dendrites of cochlear root neurons (CRNs). (A)** Parasagittal section of the cochlear nucleus complex shows labeled primary auditory afferents after injection of HRP in the basal coil of the cochlea. Notice that auditory nerve fibers divide dorsally and terminate in the dorsal part of the anteroventral cochlear nucleus (AVCN) and posteroventral cochlear nucleus (PVCN). **(A1,A2)** Higher magnification of the cochlear root nucleus corresponding to the frames in **(A)**. Note HRP-labeled endings outlining the unlabeled neuronal somata of CRNs (asterisks). **(A3,A4)** High magnification micrographs of the cochlear root nucleus show HRP-labeled collaterals of primary auditory fibers (arrowheads) that terminate on the cell body of CRNs immunolabeled for CaBP. **(B)** Parasagittal section of the cochlear nucleus complex shows labeled primary auditory afferents after injection of HRP in the apical coil of the cochlea. Notice that auditory nerve fibers bifurcate ventrally and terminate in the ventral part of the AVCN and PVCN. **(B1)** High magnification of the cochlear root nucleus corresponding to the frame in **(B)** shows HRP-labeled endings (arrowheads) outlining the unlabeled dendrite of a CRN. **(B2)** High magnification micrograph shows HRP-labeled auditory nerve endings (arrows) outlining the cell body of a CRN. **(B3,B4)** High magnification of the cochlear root nucleus shows details of HRP-labeled endings (arrowheads) outlining perpendicular **(B3)** and parallel **(B4)** unlabeled dendrites of CRNs after HRP injection in the apical coil of the cochlea. DCN, dorsal cochlear nucleus. Scale bars = 1 mm in **A** and **B**; 25μm in **A1–A4**, **B1–B4**.

To determine whether cochlear nerve terminals innervate CRNs in a tonotopic specific manner, we injected HRP into the basal and apical regions of the cochlea. In parasagittal sections of the cochlear nucleus complex, we observed HRP labeling as an intense black reaction product in which a densely bundle of axons were identified (Figure 2). These HRP labeled axons followed the characteristic V-shaped branching pattern of the primary cochlear afferents (Figures 2A,B). To define the injection site, we followed the criteria established by Merchán et al. (1988) and examined the areas of the cochlear nucleus complex in which the HRP-labeled fibers decussated and terminated. In cases with HRP injections in the basal coil of the cochlea, the cochlear nerve fibers bifurcate dorsally and give rise terminals into dorsal parts of the cochlear nucleus complex (Figure 2A). We examined the course of HRP-labeled axons within the cochlear root nucleus, and found that they give off collaterals of various diameters (ranged from 0.4 to 1.6μm) and numerous endings that terminated on CRNs somata, but not exclusively (Figures 2A1,A2). To verify that HRP-labeled fibers terminate on the cell body of CRNs, we immunostained brainstem sections for CaBP. The cochlear nerve collaterals were orientated perpendicular to the parent fibers, and extended approximately 70μm in length to innervate the cell body of CRNs immunostained for CaBP (Figures 2A3,A4). In cases with HRP injections in the apical coil of the cochlea, the cochlear nerve fibers divided more ventrally and form axonal terminals into ventral parts of the cochlear nucleus complex (Figure 2B). In the cochlear root nucleus of these cases, we observed numerous HRP-labeled fibers that span away from the CRNs cell body and followed the course of CRNs dendrites (Figure 2B1). Although we also observed that HRP-labeled fibers give off terminals on cell bodies of CRNs (Figure 2B2), a greater number of these terminals appear to establish contact on primary and distal CRNs dendrites (Figures 2B3,B4). This set of data suggests that auditory nerve inputs could be distributed preferentially on the cell body and dendrites of CRNs depending, respectively, on their basal or apical cochlear origin.

### Cochlear nerve endings on CRNs colabel with VGLUT1

**Figure 3:**
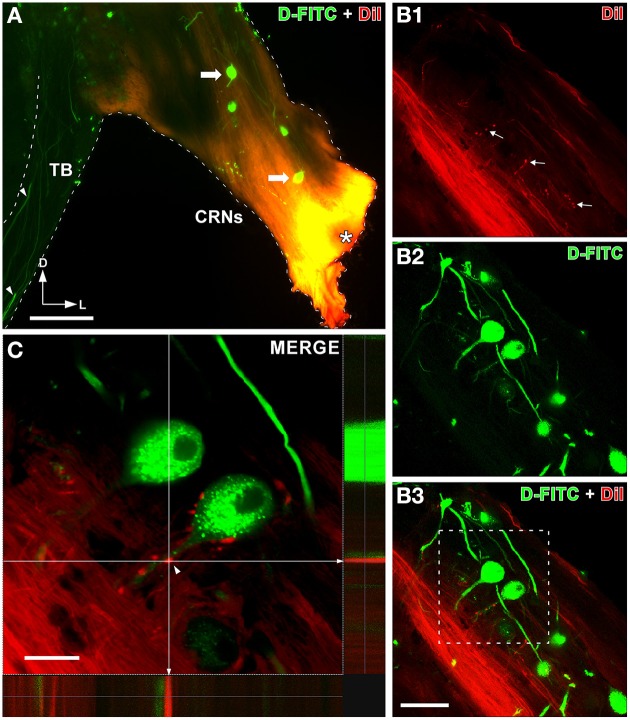
**Auditory nerve projections to cochlear root neurons (CRNs) dendrites**. **(A)** Epi-fluorescence micrograph of a coronal section shows a representative case with an insertion of DiI crystals into the cochlear root (asterisk) and retrogradely labeled CRNs (arrows) after D-FITC injection into the trapezoid body (TB). Notice thick CRNs axons (arrowheads) labeled with D-FITC coursing through the TB. **(B1–B3)** Confocal micrographs show auditory nerve terminals on dendrites of CRNs. DiI-labeled auditory nerve fibers which send collaterals (arrows) into the region of CRNs are shown in **(B1)** (in red). CRNs retrogradely labeled with D-FITC are shown in **(B2)** (in green). The image **(B3)** is the merge of the two maximum z-series projection shown in **(B1,B2)**. **(C)** Detail of the boxed area in **(B3)** show a dendrite of a CRN decorated with auditory terminals. The orthogonal view in **(C)** confirms that auditory nerve terminals are in close apposition with the dendrite (arrowhead). Scale bars = 200μm in **A**; 75μm in **B1–B3**; 25μm in **C**.

**Figure 4:**
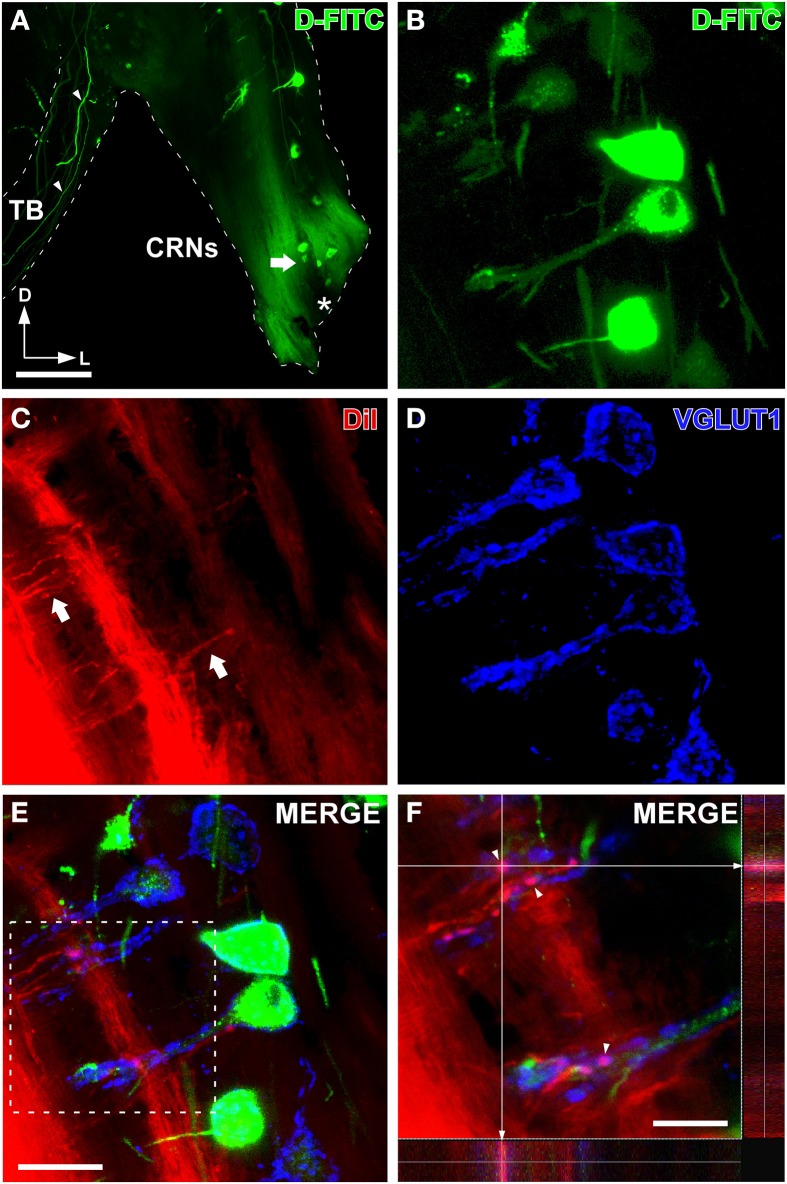
**Auditory nerve endings onto cochlear root neurons (CRNs) colabel with VGLUT1**. **(A)** Epi-fluorescence micrograph of a coronal section shows retrogradely labeled CRNs after D-FITC injection into the trapezoid body (TB) and the insertion site of DiI crystals into the cochlear root (asterisk). Notice thick CRNs axons (arrowheads) labeled with D-FITC coursing through the TB. **(B)** Confocal image of retrogradely labeled CRNs with D-FITC (position denoted by an arrow in **A**). **(C)** Confocal image of DiI-labeled endings which arise from auditory nerve collaterals (arrows). **(D)** Confocal image shows VGLUT1-immunolabeled endings. **(E)** Confocal image shows VGLUT1 colabeled with auditory nerve terminals on CRNs. This image is the merge of the three maximum z-series projection shown in **(B–D)**. **(F)** Detail of the boxed area in E shows VGLUT1-auditory nerve terminals (arrowheads) on dendrites of CRNs. Colocalization of VGLUT1 puncta and DiI is confirmed by the orthogonal view. Scale bars = 500μm in **A**; 40μm in **B–E**; 20μm in **F**.

Our HRP injections in the apical regions of the spiral ganglion gave evidence that primary auditory afferents might innervate dendrites of CRNs. To fully confirm this result, we retrogradely labeled CRNs by injecting D-FICT in the TB and inserted DiI crystals into the cochlear nerve root (Figure 3). The D-FICT injection sites in the TB were equal as those reported in our previous studies (Gómez-Nieto et al., 2008b; Gómez-Nieto and Rubio, 2009). Our D-FICT injections filled axons of approximately 3μm in diameter that coursed through the contralateral TB (Figure 3A, see also Figure 4A below). We followed the directions of these labeled axons and found that they emerged from retrogradely labeled cell bodies of CRNs (Figure 3A). The DiI crystals inserted into the cochlear nerve root led to diffusion along auditory nerve fibers (Figure 3A). In the cochlear root nucleus, we observed thin DiI-labeled collaterals branching perpendicularly from the main auditory nerve fibers (Figure 3B1). The DiI-labeled collaterals gave rise to small endings that terminated on dendrites of CRNs (Figures 3B1–B3). The orthogonal view of confocal z-stack confirmed that those cochlear nerve endings were in close apposition to dendrites of CRNs (Figure 3C).

The excitatory synaptic marker VGLUT1 has been associated with the mediation of glutamate transport at cochlear nerve fibers in other areas of the cochlear nucleus complex (Zhou et al., 2007; Gómez-Nieto and Rubio, 2009). As expected from our previous report (Gómez-Nieto et al., 2008b), we found a strong immunoreactivity for VGLUT1 in the cochlear root nucleus (Supplemental Figure 1). VGLUT1-labeled endings equally distributed throughout dorsal and ventral portions of the cochlear root nucleus (Supplemental Figure 1). They were quite numerous and fully decorated cell bodies as well as primary and distal dendrites of CRNs (Supplemental Figure 1). To determine whether these VGLUT1 immunopositive endings colabel with terminals of the auditory nerve, we used a triple-labeling method consisting of double tract-tracing with D-FITC for CRNs and DiI for cochlear nerve endings, combined with immunofluorescence for VGLUT1. As described in the above experiments, D-FITC injection in the TB retrogradely labeled the cell body and dendrites of CRNs (Figures 4A,B). The inserted DiI crystals into the cochlear nerve root diffused through cochlear nerve fibers and collaterals, allowing us to label cochlear nerve terminals (Figure 4C). We also found many VGLUT1-immunopositive endings that were outlining cell bodies and dendrites (Figure 4D). Our confocal analyses identified cochlear nerve terminals as VGLUT1-immunopositive endings that were closely apposed to CRNs (Figure 4E). The orthogonal view of cochlear nerve endings confirmed the colocalization DiI-labeled endings with VGLUT1 immunolabeling (Figure 4F).

### Electrophysiological responses of PnC neurons to contra-, ipsi-, and bilateral acoustic stimulations

**Figure 5:**
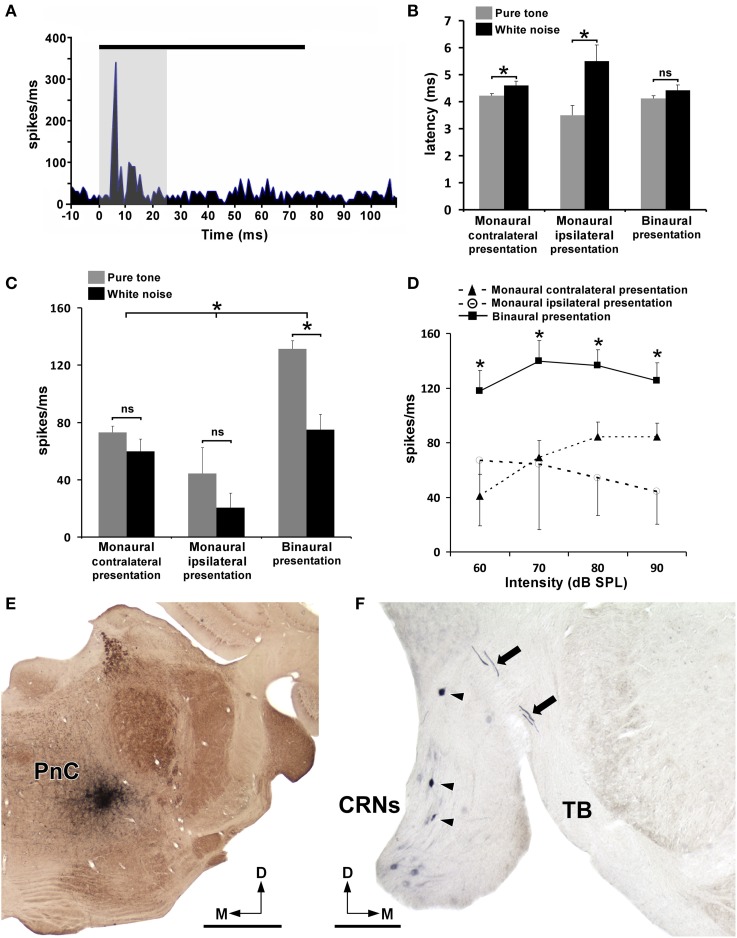
**Electrophysiological responses of PnC neurons to contra-, ipsi-, and bilateral acoustic stimulations**. **(A)** PST histogram shows the PnC neuronal activity (spikes/ms) evoked by noise bursts of 90 dB SPL and 75 ms duration (black line) delivered contralaterally to the recording site. Notice that the mean first spike latency is very short (4.39 ± 0.12 ms). The shadow area displays the integration window (first 25 ms after stimuli presentation) used to calculate the firing rate. **(B)** Histogram shows mean spike latencies of PnC neurons after contra-, ipsi-, and bilateral acoustic stimulations with noise bursts and pure tones. Notice that the spike latencies are shorter following pure tone than noise burst stimulations, with significant differences when stimuli were presented monaurally (both contra- and ipsilateral to the recording site). No significant difference (ns) was found after binaural presentations. Bars represent mean values and SEM, ^*^ [contralateral presentation, *F*_(1, 216)_ = 4.399, *p* = 0.037; ipsilateral presentation, *F*_(1, 216)_ = 8.137, *p* = 0.005]. **(C)** Histogram shows the mean firing rate (spikes/ms) of PnC neurons within the first 25 ms after contra-, ipsi-, and bilateral acoustic stimulations with noise bursts and pure tones. Binaural presentations showed significantly higher firing rates than monaural [^*^, *F*_(2, 216)_ = 14.852, *p* = 0.000]. Significant differences were found between noise bursts and pure tones following binaural presentations [^*^, *F*_(2, 216)_ = 4.037, *p* = 0.019], whereas no significant differences (ns) were found in monaural presentations. Bars represent mean values and SEM. **(D)** Firing rate-intensity function of PnC neurons in response to pure tones following contra-, ipsi-, and bilateral stimulus presentations. Binaural presentations showed significant differences with monaural presentations (both contra- and ipsilateral to the recording site) for tones of 60, 70, 80, and 90 dB SPL. Data points represent the means with SEM, [^*^, *F*_(6, 216)_ = 3.014, *p* = 0.015]. **(E)** Low magnification micrograph shows a representative case of a recording site labeled with BDA. **(F)** Micrograph shows CRNs retrogradely labeled with BDA (arrowheads) after the injection site shown in **(E)**. Notice thick BDA-labeled axons of CRNs in the trapezoid body (TB, arrows). CRNs, cochlear root neurons; PnC, caudal pontine reticular nucleus. Scale bars = 1 mm in **E**; 200μm in **F**.

To determine whether acoustically driven PnC neurons are involved in binaural summation processing, pure tones (frequency range from 0.5 to 35 kHz) and noise bursts of 75 ms duration were presented contra-, ipsi-, and bilateral to the recording site at four different intensities (60, 70, 80, and 90 dB SPL). A total of 169 and 49 multi-units recordings were obtained using pure tones and noise bursts, respectively. PnC neuronal activity exhibited an onset response with very short first spike latencies (Figure 5). For example, in Figure 5A noise bursts of 90 dB SPL evoked the PnC neuronal activity with a mean spike latency of 4.39 ± 0.12 ms. The major firing rate was observed within the first 25 ms after stimuli presentation (Figure 5A). Using this integration window, we examined the mean spike latency and firing rate of PnC neurons depending on acoustic stimuli (noise bursts and pure tones) and on the side of stimuli presentation (Figures 5B,C). We found that the mean spike latency was shorter with pure tone (3.94 ± 0.39 ms) than with noise burst stimulations (4.83 ± 0.58 ms), showing significant differences when they were presented monaurally (both contra- and ipsilateral to the recording site). However, no significant difference was found between pure tone and noise burst stimulations after binaural presentations (Figure 5B). There was also no significant difference in spike latencies when comparing binaural with monaural presentation of pure tones or noise bursts. Regarding the PnC neuronal activity, we found that the mean firing rate (spikes/ms) was significantly greater with binaural than with monaural presentations (Figure 5C). Thus, we consistently observed that either pure tones or noise bursts evoked higher responses when they were presented binaurally than when delivered monaurally. Significant differences were also found between pure tone and noise burst stimulations following binaural presentations, whereas monaural presentations showed no significant differences (Figure 5C). We further studied the neuronal activity of PnC neurons by presenting contra-, ipsi-, and bilateral pure tones at many intensities (Figure 5D). With contralateral presentations of the tones, we observed a greater increase in firing rates with increasing stimulus intensity. In contrast, this effect of the intensity was not observed with ipsilateral presentations of the tones (Figure 5D). For all the intensities tested, the firing rate was significantly greater with binaural than with monaural presentations (Figure 5D). In all the experiments, the bilateral evoked responses in PnC were almost equal to the sum of the contralateral and ipsilateral evoked responses, suggesting the existence of a binaural summation process (Figures 5C,D). To identify the origin of short-latency acoustic inputs to the acoustically driven area of PnC, we injected BDA at the end of each experiment. The BDA injections were small (~0.3 mm), round in shape, and restricted to ventrocaudal regions of the PnC (Figure 5E, see also Supplemental Figures 2, 3). In all cases, we found BDA-labeled axons in the TB that emerged from retrogradely labeled CRNs (Figure 5F). The number of BDA-labeled neurons located on the contralateral cochlear root nucleus (75.6%) was considerably higher than on the ipsilateral nucleus (24.4%). This result indicates that CRNs provide short latency acoustic inputs to PnC with a clear contralateral preference. Because BDA is an effective bidirectional pathway tracer (Rajakumar et al., 1993), we also observed retrogradely labeled neurons and anterogradely labeled terminals in the PnC contralateral to the recording/injection site (Supplemental Figures 2, 3). The labeled fibers from PnC neurons of the right side crossed the midline and enter into the opposite (left) reticular formation giving off BDA-labeled terminals onto retrogradely labeled PnC neurons (Supplemental Figures 2, 3). This result supports the idea that reciprocal connections might exist between the left and right PnC. In cases with slightly larger injections, we also found very few neurons retrogradely labeled by BDA in the DCN and VCN (Supplemental Figure 4). However, the number of retrogradely labeled neurons in the DCN and VCN was considerably less than those labeled in the cochlear root nucleus (see table in Supplemental Figure 4).

### BDA injections in the dorsal and ventral cochlear nucleus

**Figure 6:**
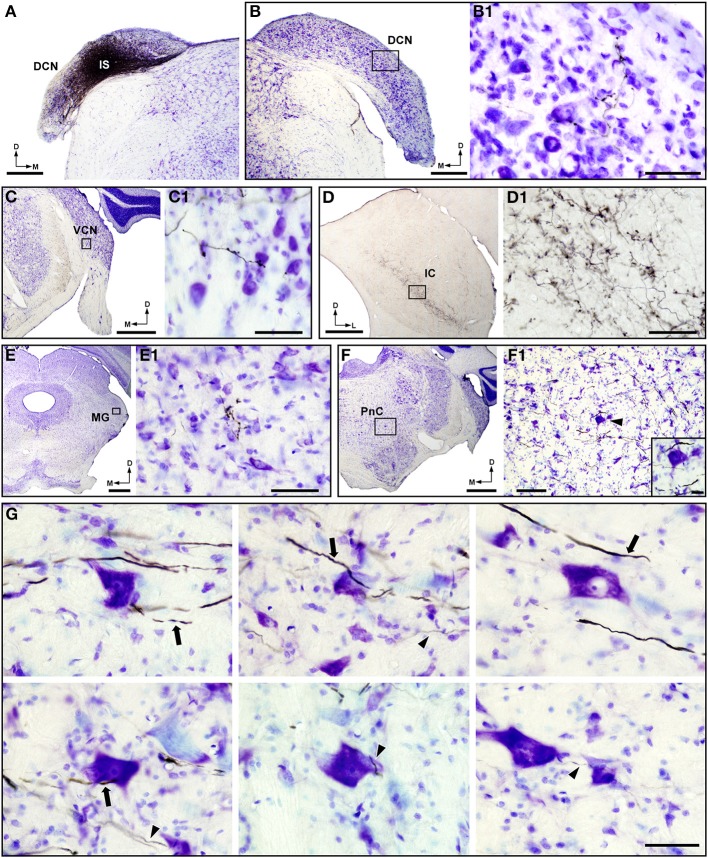
**BDA injections in the dorsal cochlear nucleus (DCN) do not generate labeled terminals on giant neurons of the caudal pontine reticular nucleus (PnC)**. **(A)** Micrograph of a Nissl-stained coronal section shows a BDA injection site (IS) in the DCN. **(B–E)** Micrographs of the contralateral DCN **(B)**, the contralateral ventral cochlear nucleus (VCN; **C**), the contralateral inferior colliculus (IC; **D**) and the contralateral medial geniculate body (MG; **E**) show anterograde labeling after the BDA injection shown in **(A)**. (**B1–E1)** Higher magnification corresponding to the frames in **(B–E)** respectively, shows details of BDA-labeled terminals in those nuclei. **(F)** Nissl-stained section of the contralateral PnC shows axons labeled with BDA crossing the pontine reticular formation (see higher magnification in **F1**). The **F1** inset (position denoted by an arrowhead) shows the absence of labeled terminals on a giant PnC neuron. **(G)** High magnification micrographs show representative examples of thick and thin BDA-labeled axons (denoted by arrows and arrowheads, respectively) crossing the caudal pontine reticular formation. Notice that DCN projecting axons do not give off endings onto Nissl-stained PnC neurons. Scale bars = 500μm in **A** and **B**; 50μm in **B1**, **C1**, **D1**, **E1**, and **G**; 1 mm in **C–F**; 200μm in **F1**; 25μm in the **F1** inset.

**Figure 7:**
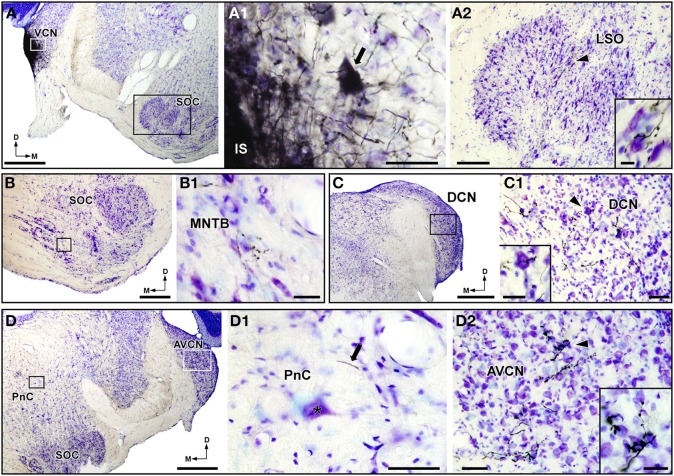
**BDA injections in the ventral cochlear nucleus (VCN) do not generate labeled terminals on giant neurons of the caudal pontine reticular nucleus (PnC)**. **(A)** Micrograph of a Nissl-stained coronal section shows a case with BDA injections in the VCN. **(A1)** A higher magnification (corresponding to the white frame in **A**) shows a labeled VCN projection neuron (arrow) in the proximity of the BDA injection sites (IS). **(A2)** Nissl-stained section of the ipsilateral lateral superior olive (LSO), corresponding to the black frame in **(A)**, shows the characteristic projection pattern of VCN neurons. The inset in **(A2)** (position denoted with an arrowhead) illustrates a higher magnification of BDA-labeled terminals in the LSO. **(B,C)** Micrographs of the contralateral medial nucleus of the trapezoid body (MNTB; **B**) and the contralateral dorsal cochlear nucleus (DCN; **C**) show anterograde labeling after the BDA injections shown in **(A)**. **(B1–C1)** Higher magnification of BDA-labeled terminals corresponding to the frame in **(B,C)**, respectively. The inset in **(C1)** (position denoted with an arrowhead) illustrates a higher magnification of BDA-labeled terminals on a Nissl-stained DCN neuron. **(D)** Micrograph of a Nissl-stained section containing the contralateral PnC and the anteroventral cochlear nucleus (AVCN). **(D1)** High magnification (corresponding to the black frame in **D**) shows the absence of labeled terminals on PnC neurons (asterisk). Note that labeled fibers (arrow) do not give off terminals. **(D2)** High magnification (corresponding to the white frame in **D**) shows anterograde labeling in the contralateral AVCN. The inset in **(D2)** (position denoted with an arrowhead) illustrates details of BDA-labeled terminals on Nissl-stained AVCN neurons. SOC, superior olivary complex. Scale bars = 1 mm in **A**, **C**, and **D**; 50μm in **A1**, **C1**, **D1**, and **D2**; 200μm in **A2**; 10μm in **A2** inset; 500μm in **B**; 25μm in **B1** and insets of **C1** and **D2**.

The results described above indicated that acoustically driven PnC neurons receive bilateral, but mainly contralateral, inputs from CRNs. However, the presence of very few retrogradely labeled neurons in other areas of the cochlear nucleus complex led us to investigate whether these regions innervate PnC neurons. This was done by injecting BDA into the left DCN and VCN. All injections sites of BDA in the DCN and VCN were restricted to the corresponding nucleus and did not spread to adjacent areas of the cochlear nucleus complex (Figures 6A, 7A). BDA injections in the DCN were small (0.5–0.7 mm in diameter) and round in shape (Figure 6A). To check the efficiency of BDA injections in the DCN, we analyzed the distribution of anterograde labeling in nuclei that are well known to receive DCN inputs (Cant and Gaston, 1982; Malmierca et al., 2002; Cant and Benson, 2003). Our material showed BDA-labeled fibers and terminals in the contralateral DCN (Figure 6B), the ipsilateral (data not shown) and contralateral VCN (Figure 6C), the contralateral IC (Figure 6D), and the contralateral medial geniculate body (Figure 6E). We also observed thin and thick labeled fibers of approximately 0.7 and 2.8μm in diameter, respectively, in the pontine reticular formation (Figures 6F,G). We examined both type of BDA-labeled fibers and found that they do not innervate giant PnC neurons (Figure 6G). Due to the bidirectional nature of the transport of the BDA, the cochlear nerve terminals that innervate DCN neurons uptake the tracer and filled retrogradely cochlear nerve fibers (Supplemental Figure 5). We followed these retrogradely labeled fibers and found that they innervate CRNs somata in a similar pattern than that observed in our HRP injections in the basal coil of the cochlea (for comparisons see Figures 2A1–A4 and Supplemental Figure 5). In cases with BDA injection in the VCN, we obtained small (0.3–0.8 mm in diameter) and slightly elongated injection sites (e.g., in Figure 7A). These injections generated anterograde labeling in nuclei that are known to be targeted by VCN projecting neurons (Cant and Gaston, 1982; Friauf and Ostwald, 1988; Doucet and Ryugo, 1997, 2003; Cant and Benson, 2003). Thus, we observed a thin band of axons and swellings in the ipsilateral lateral superior olive (Figure 7A2) as well as BDA-labeled terminals in the contralateral medial nucleus of the TB (Figure 7B), the contralateral DCN (Figure 7C), and the contralateral anteroventral cochlear nucleus (Figure 7D2). In the reticular pontine formation, we found BDA-labeled fibers that passed in close proximity to giant PnC neurons, but without giving off any terminal fields (Figure 7D1). In sum, these track-tracing experiments suggest that other areas of the cochlear nucleus complex are not likely to innervate PnC neurons.

### The acoustic startle reflex in rats with monaural sound-deprivation

**Figure 8:**
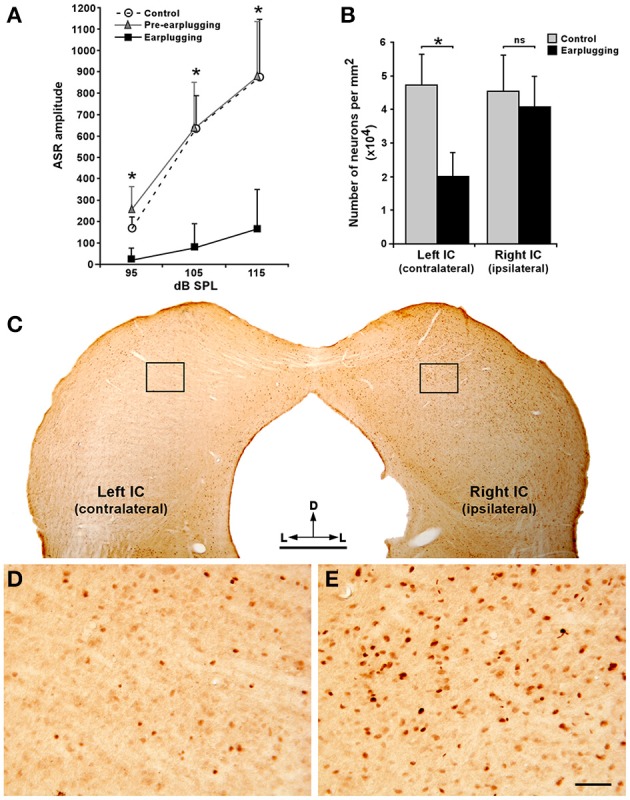
**The acoustic startle reflex (ASR) in rats with monaural sound-deprivation**. **(A)** Amplitude-intensity function of the ASR in response to noise bursts shows differences between the control (*n* = 6) and the experimental group (*n* = 6), before and following monaural earplugging. Notice that ASR amplitude was significantly higher in control and pre-earplugging rats than earplugged animals at all intensities tested (95, 105, and 115 dB SPL). Data is expressed in mean values and error bars represent standard deviation (SEM), ^*^ *p* < 0.05 [control vs. monaural earplugged animals, *F*_(1, 12)_ = 6.32; *p* = 0.04; experimental group, pre-earplugging vs. monaural earplugging, *F*_(5, 6)_ = 8.45, *p* = 0.008]. The source of variability is the number of animals and trials. **(B)** Histogram shows the number of c-Fos immunopositive neurons per mm^2^ in the inferior colliculus (IC) of control and monaural earplugged animals. Notice that the contralateral plugged side (left IC) contains significantly less neurons than the left IC of control animals. However, the right IC of control and monaural earplugged animals showed no significant differences (ns). Bars represent mean values and SEM, ^*^p < 0.05. The variability observed in the stereological study comes from sampling one section containing the left and right IC in each one of the 12 animals used in the behavioral experiments**. C**, Low magnification micrograph shows c-Fos immunoreactivity in the IC of a monaural earplugged animal. **(D,E)** High magnification micrographs corresponding to the frames in **(C)** show c-Fos immunolabeling in the contralateral (**D**, left IC) and ipsilateral plugged side (**E**, right IC). Note the number of c-Fos immunolabeled neurons is considerable less in the contralateral than the ipsilateral plugged side. Scale bars = 1 mm in **C**; 50μm in **D** and **E**.

Our electrophysiological and morphological studies indicated the existence of a binaural summation process in the neuronal activity of PnC neurons that receive acoustic inputs from CRNs. To determine whether these results are consistent with the binaural summation of the behavioral response, we investigated the ASR in rats prior to and following monaural earplugging. By plugging the sound reaching from one ear, the acoustic inputs to the CRNs in the right was reduced, and hence affected the bilateral afferent processing within the ASR pathway. Changes of the ASR amplitude for each intensity of the stimulus (noise bursts of 95, 105, and 115 dB SPL) are shown in Figure 8A and Supplemental Figure 6. In general, an increase of the acoustic stimulus intensity resulted in a proportional increase of the ASR amplitude. As compared to controls animals, we observed that the ASR amplitude was significantly lower in monaural earplugged rats [*F*_(1,12)_ = 6.32; *p* = 0.04]. A similar reduction was found when compared the experimental group prior to and following monaural earplugging, showing significant differences in the tested range of stimulus intensity [*F*_(5,6)_ = 8.45; *p* = 0.008]. On the contrary, there were no significant differences in ASR amplitude between control and pre-earplugged animals (Figure 8A). We also found no significant differences in ASR latency between normal hearing (control and pre-earplugged animals) and monaural earplugged conditions at all stimulus intensities tested (Supplemental Figure 6). As a histological control, c-Fos immunoreactivity in the IC was assessed to check the efficiency of the monaural earplugging (Figures 8B–E). In the contralateral plugged side, the number of c-Fos immunolabeled neurons was significantly less in earplugged rats than in controls (*p* < 00.5), whereas a similar number of neurons were observed in the ipsilateral side (Figure 8B). When the ipsi- and contralateral sides to the earplugging were compared, the contralateral side showed a comparative largest decrease in c-Fos immunolabeling (Figures 8C–E). This indicates that the earplug attenuated the sound from reaching the right ear, and hence, reduced the neuronal activity of the contralateral auditory pathway including the left IC (Figures 8C–E). In sum, we showed that the ASR amplitude was higher when the acoustic startling stimulus was processed binaurally than when it was processed monaurally, indicating that there is a strong summation for ASR. Comparing the electrophysiological and behavioral experiments, we found that the reduction of PnC neurons' responses after monaural acoustic stimulation is consistent with the reduction in the ASR amplitude following the monaural sound-deprivation (for comparisons see Figures 5D, 8A).

## Discussion

In this study, we showed key morphofunctional aspects of the ASR primary neuronal circuit. In the first central relay station, we found that dendrites and cell bodies of CRNs receive selective inputs from specific regions of the cochlea. We also verified that these cochlear nerve inputs are immunopositive for VGLUT1 as occurs in other areas of the cochlear nucleus complex (Zhou et al., 2007; Gómez-Nieto and Rubio, 2009). In the second central relay station, we demonstrated that the short-latency acoustic inputs to PnC neurons are provided mainly, if not exclusively, by CRNs. Our electrophysiological results indicated that there is a strong binaural summation in the neuronal activity of PnC neurons which can be linked to the bilateral projections from the cochlear root nucleus to the PnC (López et al., 1999; Nodal and López, 2003). Finally and accordingly with our electrophysiological findings, we showed that the overall response of the behavioral paradigm is higher when the acoustic startling stimulus is processed binaurally than when processed monaurally. Our study clearly supports that the functional connections of the cochlear root nucleus with PnC constitutes the neuronal bases underlying the rapid short-latency and binaural summation of the ASR.

### Tonotopic-specific distribution through the cell body and dendrites of CRNs

Alerting and escape behaviors have high dependence on the frequency of sounds, which are of great importance in the life of rodents. For example, rats emit ultrasounds in the frequency range between 18 and 30 kHz in response to a threatening stimulus (predator exposure), and hence, serve as alarm calls to specifically warn other individuals (Cuomo and Cagiano, 1987; Blanchard et al., 1991). CRNs provide acoustic information to a wide variety of non-auditory nuclei involved in the startle reflex, orientation of head and ears toward a novel sound, vocalization, emotional information, and escape (López et al., 1999; Nodal and López, 2003; Horta-Júnior et al., 2008). Since these sensory events and escape behaviors are initially mediated by CRNs (Lee et al., 1996; López et al., 1999), it is likely that CRNs receive selective inputs from specific regions of the cochlea. Our HRP injections in apical and basal coils of the cochlea indicated that the cell body and dendrites of CRNs are preferentially innervated by different portions of the cochlea. Fibers from basal, high frequency parts of the cochlea, terminated preferentially on the cell body whereas those from more apical, low frequency parts of the cochlea, mainly terminated on dendrites. These qualitative observations are in agreement with Osen et al. (1991) studies, which showed that the base of the cochlea is the source of most primary inputs to CRNs somata. Our study further demonstrated that dendrites of CRNs, which are quite extensive, are highly innervated by cochlear nerve inputs. The technical challenge of labeling dendrites of CRNs while analyzing the primary afferents might explain why this result was not shown in our previous reports (Merchán et al., 1988; Osen et al., 1991; López et al., 1993). In the present study, the experimental approach designed by Gómez-Nieto and Rubio (2009) combining two neuronal tracers, D-FICT in the TB and DiI in the cochlear nerve root, allowed us to confirm that CRNs dendrites also receive cochlear nerve inputs. The frequency-specific distribution described in our study provides an explanation for the frequency tuning curves observed in extracellular recordings of CRNs (Sinex et al., 2001; Gómez-Nieto et al., 2013). Thus, our results showed that CRNs somata and primary dendrites receive massive high-frequency inputs that might be responsible for the high characteristic frequency of CRNs (~30 kHz). Since the basal regions of the cochlea have also the shortest latencies, these might provide a specialization to minimize the response latency in CRNs (Sinex et al., 2001). Furthermore, it is interesting to point out that CRNs responded to a frequency range from 1.5 to 40 kHz that is nearly the entire audiogram of the rat (Kelly and Masterton, 1977; Sinex et al., 2001), which is in line with our observations showing high and mostly low frequency inputs on CRNs dendrites. Our data together with the fact that the synaptic strength becomes larger as one moves along the dendrite (Spruston, 2000; London and Segev, 2001) suggest that CRNs, which are high frequency specialized neurons, might also integrate a wide range of frequencies in their dendrites.

### Rapid glutamatergic transmission in the cochlear root nucleus

The ASR behavioral paradigm is defined by its rapid and short latency reflex actions (Szabo, 1964; Hoffman and Ison, 1980). For that, CRNs have specific morphological, physiological and neurochemical properties that provide them the capacity of mediating fast and secure neurotransmission of short-latency auditory cues (López et al., 1993, 1999; Sinex et al., 2001). An important outcome of our study was to demonstrate that cochlear nerve inputs on CRNs colabeled with VGLUT1. This result together with the fact that VGLUT1 terminals massively covered CRNs on the z axis (Gómez-Nieto et al., 2008b; see also our results) indicates that auditory nerve terminals on CRNs are far more numerous than reported in previous studies (Merchán et al., 1988). Since cochlear nerve fibers use glutamate as neurotransmitter (Hackney et al., 1996; Rubio and Juiz, 2004; Rubio, 2006), VGLUT1 might contribute to the synaptic efficacy by regulation of vesicle cycling and filling (Wilson et al., 2005). Furthermore, the relatively low affinity for their substrate allows the VGLUT1 to transport large amounts of glutamate more rapidly (Bergles and Edwards, 2008). Thus, our results showed that CRNs somata and dendrites are fully covered by VGLUT1-cochlear nerve terminals which provide a great speed on synaptic signaling in the first component of the ASR circuit. This VGLUT1-cochlear nerve colabeling has been also found on bushy cells of the VCN (Gómez-Nieto and Rubio, 2009), a neuronal type that encodes features of the acoustic waveform and conveys precise temporal information to upper auditory structures (Friauf and Ostwald, 1988; Cant and Benson, 2003). VGLUT1 terminals on CRNs (ASR pathway) and bushy cells (auditory pathway) exhibit an altered morphology in mice with targeted deletion of the gene coding for the auxiliary subunit α_2_δ 3 of voltage-gated calcium channels (Pirone et al., 2014). Both neuronal types express the α_2_δ 3 subunit and its lack lead to neuronal deficits in the auditory and the ASR pathway, including the inability to discriminate temporal dimensions of sounds (Pirone et al., 2014). Thus, CRNs might provide fast acoustic information with accurate temporal precision to the ASR pathway just as bushy cells do for the ascending auditory pathway. This idea is in line with our findings that support the CRNs-PnC projection as the neuronal pathway underlying the binaural summation of the ASR (discussed below).

### Origin of short-latency acoustic inputs to PnC neurons

A conclusive demonstration that one nucleus is not innervated by another is difficult to accomplish in any region of the central nervous system. This became more difficult if that nucleus, as occurs in PnC, receives a large number of inputs and contains numerous crossing fibers. Bearing this argument in mind, our study provided evidence that supports the CRNs as the solely source of short-latency acoustic inputs to giant PnC neurons. Our electrophysiological experiments with subsequent neuronal tracing showed retrogradely labeled CRNs after recording acoustically driven PnC neurons. This result is consistent with our previous reports that demonstrated the projections from the cochlear root nucleus to PnC neurons (López et al., 1999; Nodal and López, 2003). In addition, our BDA injections in PnC also generated very few retrogradely labeled neurons in the DCN and VCN, which in principle suggests that other areas of cochlear nucleus complex might provide short-latency acoustic inputs to PnC as proposed by other studies (Davis et al., 1982; Kandler and Herbert, 1991; Lingenhöhl and Friauf, 1994; Meloni and Davis, 1998). Lingenhöhl and Friauf (1994) reported retrogradely labeled somata in DCN and VCN after injecting a pure retrograde trace (FluoroGold) in PnC. By contrast, our restricted BDA injections into the DCN and VCN were unable to demonstrate the connection of these two areas with giant PnC neurons. Although we filled a great number of DCN and VCN projecting neurons, as shown by the labeled terminal fields in many nuclei known to receive innervations from the DCN and VCN (reviewed in Cant and Benson, 2003), we did not find any terminal fields on PnC neurons. A possible explanation for these contradictory results is that DCN and VCN neurons were retrogradely labeled after tracer injections in PnC as a consequence of the tracer uptake by fibers of passage rather than by terminals on PnC neurons. This idea seems to be consistent with previous studies reporting that DCN and VCN projections to PnC were very weak, and not as dense as those from the cochlear root nucleus (Friauf and Ostwald, 1988; Kandler and Herbert, 1991; Meloni and Davis, 1998). In accordance with this morphological data, behavioral studies showed that chemical lesions of CRNs drastically reduced the startle response at all intensities (Lee et al., 1996), whereas electrolytic lesions of the DCN did not (Davis et al., 1982; Meloni and Davis, 1998). Meloni and Davis (1998) found that electrolytic lesions of the DCN lead to a significant reduction in ASR amplitude at 110 and 115 dB SPL startle-eliciting intensities and normal responses on all other intensities. Interestingly, one finding of our BDA injections in DCN was that some auditory nerve terminals on CRNs and DCN neurons arise from the same parent auditory nerve fiber. According to this, electrolytic lesions of the DCN might damage auditory nerve terminals on CRNs, and might reduce the ASR amplitude at high intensities. It is well established that auditory nerve fibers that reach the dorsal part of the cochlear nucleus complex originated from the basal coils (high-frequency) of the cochlea (Saint Marie et al., 1999). Thus, our BDA injections in the dorsal part of the DCN generated retrograde labeled terminals on the cell body of CRNs in a similar pattern than that observed in our HRP injections in the basal coil of the cochlea. This result verifies the tonotopic-specific distribution through the cell body and dendrites of CRNs and suggests that both CRNs and DCN neurons receive similar acoustic information. DCN projects to the IC (Beyerl, 1978; Oliver and Shneiderman, 1991; Oliver et al., 1999; Cant and Benson, 2003), which is known to participate in the modulation of the ASR (Leitner and Cohen, 1985; Fendt et al., 2001; Yeomans et al., 2006; Gómez-Nieto et al., 2008a, 2013). It is, therefore, likely that DCN might provide acoustic information to ASR modulation pathways rather than being necessary for the initiation and elicitation of the ASR.

### Neuronal bases underlying binaural summation of the acoustic startle reflex

Our electrophysiological data showed that PnC neurons, which are innervated by CRNs, responded with very short spike latencies to noise bursts. The fact that sounds that contain every frequency activate PnC neurons is consistent with our hypothesis that frequency integration occurs in CRNs dendrites, and implies that CRNs provide precise and rapid acoustic information to PnC neurons. Our previous studies reported that CRNs have thick myelinated axons and contain calcium binding proteins that confer to the cochlear root nucleus the necessary specializations for sending fast electric signals to PnC (López et al., 1993, 1999). Accordingly, more recent studies have demonstrated that proteins such as the potassium channel subunit Kv1.1 and the transcription factor Math5-lacZ are highly expressed in CRNs (Oertel et al., 2008; Saul et al., 2008). These proteins have been found to participate in fast neurotransmission, temporal synchrony, and processing of binaural information (Saul et al., 2008; Allen and Ison, 2012). Consistently with the molecular specializations of CRNs, an important conclusion that we draw from our electrophysiological experiments is that the CRNs-PnC projections determine the binaural summation of the ASR. This phenomenon of binaural summation provides that the startling stimulus presented to both ears is perceived as more intense than if it were presented in monaural mode. Therefore, there is strong binaural summation for startle, with a preference for acoustic stimuli delivered near the midline to activate both ears simultaneously (reviewed in Yeomans et al., 2002). Accordingly, we showed that the binaural evoked responses of PnC neurons were almost the sum of those evoked monaurally. The fact that the bilateral CRNs-PnC projections have a clear contralateral predominance (López et al., 1999; Nodal and López, 2003) might explain our result showing higher PnC neurons' responses evoked with contralateral than ipsilateral acoustic stimulations. Our electrophysiological analysis also revealed that PnC neurons' responses increased as the contralateral stimulus intensity increases, a result which is in line with our behavioral data and the contralateral predominance of the CRNs-PnC pathway. Wagner et al. (2000) suggested that the superior olivary complex, which is involved in binaural processing, is necessary for the full expression of the ASR. However, the possibility that neuronal circuits beyond the cochlear nucleus complex contribute to the binaural summation of the ASR seems very limited. The ASR mediated via the superior olivary complex involves too many synapses to accomplish the short latency of the ASR, and this led us to restrict the binaural summation to the CRNs-PnC pathway. In accordance with two reports in rats (Lingenhöhl and Friauf, 1994) and cats (Walberg, 1974), our study suggests the existence of reciprocal connections between the left and right PnC, which need to be accounted for the final evoked response of PnC neurons. Further research is required to learn more about these crossed reticulo-reticular connections and their possible functional role in the ASR. It is also relevant to note that our electrophysiological results were consistent with our behavioral experiments showing that the ASR amplitude was higher when the acoustic startling stimulus was processed binaurally than when it was processed monaurally. We came to this conclusion comparing the behavioral response of the control animals with that of monaural earplugged animals. Therefore, it was essential in this experimental design to verify the effectiveness of the earplugging. Since c-Fos protein has been widely used as a marker of early neuronal activation (Sagar et al., 1988; Dragunow and Faull, 1989; Murphy and Feldon, 2001), we quantified the c-Fos immunolabeling in the IC. The IC was selected for c-Fos quantification because it is an obligatory relay center for most ascending auditory tracts (Beyerl, 1978; Oliver and Shneiderman, 1991) and plays an important role in the prepulse inhibition of the ASR (Leitner and Cohen, 1985; Fendt et al., 2001; Li and Yue, 2002; Yeomans et al., 2006; Gómez-Nieto et al., 2008a, 2013). Our results showed that the number of c-Fos immunolabeled neurons in the contralateral IC was drastically reduced by the earplugging. This reduction was not found in the ipsilateral side to the earplugging because the IC receives auditory inputs from the contralateral cochlear nucleus (Beyerl, 1978; Oliver and Shneiderman, 1991). Since monaural conductive hearing loss reduces auditory nerve activity and affects sound processing along the central auditory pathway (Potash and Kelly, 1980), the reduction of c-Fos immunoreactivity in the IC indicates that bilateral afferent processing within the ASR pathway was affected by the earplugging. As expected by Davis and Wagner (1969) report, we found that the ASR amplitude increased with increasing stimulus intensity in control and pre-earplugged animals. In contrast, earplugged animals showed much less ASR amplitude, suggesting that the binaural ASR pathway is required to elicit a full startling response. Our study suggests the CRNs-PnC pathway as an anatomical and physiological specialization that determines the binaural summation of the ASR. Since CRNs also projects to non-auditory nuclei involved in other startling reflex modalities (López et al., 1999; Horta-Júnior et al., 2008), it is reasonable to propose that the CRNs projections might also participate in cross-modal summation of the ASR (Yeomans et al., 2002). In conclusion, our study consolidates the CRNs as the “early warning system” responsible for the execution and propagation of bilateral acoustic startling signals at very short latencies, and that is what defines the ASR in itself.

### Conflict of interest statement

The authors declare that the research was conducted in the absence of any commercial or financial relationships that could be construed as a potential conflict of interest.

## Supplementary material

Supplemental Figure 1 **Distribution of VGLUT1-immunolabeled endings in the cochlear root nucleus. (A)** Epi-fluorescence micrograph of a coronal section shows VGLUT1-immunolabeled endings (Cy3 fluorochrome) in the cochlear root nucleus. **(B–E)** Epi-fluorescence micrographs of the boxed areas in **(A)** show distribution of VGLUT1-immunolabeled endings from dorsal to ventral regions of the cochlear root nucleus. Note that numerous VGLUT1-immunolabeled endings decorate unlabeled cell bodies (arrows) and dendrites (arrowheads) of cochlear root neurons (CRNs). TB, trapezoid body. Scale bars = 200μm in **A**; 25μm in **B–E**.

Supplemental Figure 2 **BDA injections in the caudal pontine reticular nucleus (PnC) generate anterograde and retrograde labeling in the contralateral PnC. (A)** Low magnification micrograph of a coronal section of the brainstem shows a representative case with BDA injection sites (IS) in the right PnC. The tracer was injected after recording of acoustically driven PnC neurons. Notice numerous BDA-labeled axons (arrowheads) crossing the midline toward the contralateral PnC. Also, thick axons of cochlear root neurons (position denoted with an arrow) were retrogradely labeled with BDA in the trapezoid body (TB). **(B)** Higher magnification corresponding to the frame in **(A)** shows details of BDA-labeled terminals onto retrogradely labeled giant PnC neurons (arrows). Scale bars = 1 mm in **A**; 200μm in **B**.

Supplemental Figure 3 **Reciprocal connections between the left and right caudal pontine reticular nuclei (PnC). (A)** Micrograph of a Nissl-stained coronal section shows a BDA injection site (IS) in the right PnC. The tracer was injected after recording of acoustically driven PnC neurons. Arrowheads indicate crossed reticulo-reticular projections labeled with BDA. Also, thick axons of cochlear root neurons (position denoted with an arrow) were retrogradely labeled with BDA in the trapezoid body (TB). **(B,C)** Higher magnification corresponding to the frames in **(A)** shows details of BDA-labeled terminals (arrowheads) onto Nissl-stained giant PnC neurons. Notice a PnC neuron retrogradely labeled by BDA (asterisk in **C**). Scale bars = 1 mm in **A**; 25μm in **B** and **C**.

Supplemental Figure 4 **Retrograde labeling in the coclear nucleus complex after BDA injections in the acoustically driven area of the caudal pontine reticular nucleus (PnC). (A)** High magnification micrograph shows a coronal section of the contralateral dorsal cochlear nucleus (DCN) in which a BDA-retrogradely labeled neuron (arrow) was found after the injection site shown in Supplemental Figure 2A. **(B)** High magnification micrograph shows a BDA-retrogradely labeled neuron (arrow) in the contralateral ventral cochlear nucleus (VCN). **(C)** Quantitative distribution of retrogradely labeled neurons in the cochlear nucleus complex after BDA injections in the acoustically driven area of PnC. The table shows the retrograde labeling obtained in the seven cases used in the electrophysiological experiments. Notice that the number of retrogradely labeled neurons in the DCN and VCN is considerably less than those labeled in the cochlear root nucleus. Scale bar = 200μm in **A** and **B**.

Supplemental Figure 5 **BDA injections in the dorsal cochlear nucleus (DCN) generate retrogradely labeled terminals on the cell body of cochlear root neurons (CRNs). (A)** Nissl-stained coronal section of the cochlear root nucleus shows retrogradely labeled fibers after the BDA injections shown in Figure 6A. **(B)** Higher magnification corresponding to the frame in A shows details of BDA-labeled terminals (arrowheads) on CRNs somata. Notice that the morphology and distribution of these BDA-labeled terminals resemble those of the cochlear nerve after HRP injections in the basal coil of the cochlea (see Figures 2A1–A4). VCN, ventral cochlear nucleus. Scale bars = 400μm in **A**; 50μm in **B**.

Supplemental Figure 6 **ASR amplitude, ASR latency and weight in rats prior to and following monaural earplugging. (A)** Histograms show the ASR amplitude in response to noise burst of 95, 105, and 115 dB SPL in rats before and following 4 days of monaural earplugging. Notice that ASR amplitude was significantly higher in normal hearing than following monaural earplugging at all intensities tested (95, 105, and 115 dB SPL). Data is expressed in mean values and error bars represent standard deviation (SEM), ^*^*p* < 0.05 [experimental group, pre-earplugging vs. monaural earplugging, *F*_(5,6)_ = 8.45, *p* = 0.008]. **(B)** Data-set tables display the ASR amplitude (mean values of *V*_max_ within the testing session), ASR latency (mean values of *T*_max_ within the testing session) and weight of each animal in normal hearing conditions (pre-earplugging) and following monaural earplugging. There were no significant differences in the ASR latency between the normal hearing and earplugged conditions for all stimulus intensities tested. Notice the animals maintained their weight steady throughout the behavioral experiment.

## References

[B1] Allen P. D. Ison J. R. (2012). Kcna1 gene deletion lowers the behavioral sensitivity of mice to small changes in sound location and increases asynchronous brainstem auditory evoked potentials but does not affect hearing thresholds. J. Neurosci. 32, 2538–2543 10.1523/JNEUROSCI.1958-11.2012 22396426 PMC3297021

[B2] Bergles D. E. Edwards R. H. (2008). The role of glutamate transporters in synaptic transmission, in Structural and Functional Organization of the Synapse, eds Hell W. Ehlers M. D. (New York, NY: Springer), 23–61 10.1007/978-0-387-77232-5_2

[B3] Beyerl B. D. (1978). Afferent projections to the central nucleus of the inferior colliculus in the rat. Brain Res. 145, 209–223 10.1016/0006-8993(78)90858-2 638786

[B4] Blanchard R. J. Blanchard D. C. Agullana R. Weiss S. M. (1991). Twenty-two kHz alarm cries to presentation of a predator, by laboratory rats living in visible burrow systems. Physiol. Behav. 50, 967–972 10.1016/0031-9384(91)90423-L 1805287

[B5] Błaszczyk J. W. Tajchert K. (1997). Effect of acoustic stimulus characteristics on the startle response in hooded rats. Acta Neurobiol. Exp. (Wars). 57, 315–321 9519548 10.55782/ane-1997-1240

[B6] Braff D. L. Geyer M. A. (1990). Sensorimotor gating and schizophrenia: human and animal model studies. Arch. Gen. Psychiatry 47, 181–188 10.1001/archpsyc.1990.01810140081011 2405807

[B7] Caeser M. Ostwald J. Pilz P. K. D. (1989). Startle responses measured in muscles innervated by facial and trigeminal nerves show common modulation. Behav. Neurosci. 103, 1075–1081 2803555 10.1037//0735-7044.103.5.1075

[B8] Cant N. B. Benson C. G. (2003). Parallel auditory pathways: projection patterns of the different neuronal populations in the dorsal and ventral cochlear nuclei. Brain Res. Bull. 60, 457–474 10.1016/S0361-9230(03)00050-9 12787867

[B9] Cant N. B. Gaston K. C. (1982). Pathways connecting the right and left cochlear nuclei. J. Comp. Neurol. 212, 313–326 10.1002/cne.902120308 6185548

[B10] Castellano O. Arji M. Sancho C. Carro J. Riolobos A. S. Molina V. (2013). Chronic administration of risperidone in a rat model of schizophrenia: a behavioural, morphological and molecular study. Behav. Brain Res. 242, 178–190 10.1016/j.bbr.2012.12.036 23291154

[B11] Castellano O. Moscoso A. Riolobos A. S. Carro J. Arji M. Molina V. (2009). Chronic administration of risperidone to healthy rats: a behavioural and morphological study. Behav. Brain Res. 205, 488–498 10.1016/j.bbr.2009.08.002 19665494

[B12] Cuomo V. Cagiano R. (1987). Ultrasonic vocalization in rodents: a new potential tool for detecting emotional and motivational changes produced by adverse treatments. Zentralbl. Bakteriol. Mikrobiol. Hyg. B. 185, 55–60 3124389

[B13] Davis M. (1990). Animal models of anxiety based on classical conditioning: the conditioned emotional response (CER) and the fear-potentiated startle effect. Pharmacol. Ther. 47, 147–165 10.1016/0163-7258(90)90084-F 2203068

[B14] Davis M. Gendelman D. S. Tischler M. D. Gendelman P. M. (1982). A primary acoustic startle circuit: lesion and stimulation studies. J. Neurosci. 2, 791–805 7086484 10.1523/JNEUROSCI.02-06-00791.1982 PMC6564345

[B15] Davis M. Sheard M. H. (1974). Habituation and sensitization of the rat startle response: effects of raphe lesions. Physiol. Behav. 12, 425–431 10.1016/0031-9384(74)90120-6 4820138

[B16] Davis M. Wagner A. R. (1969). Habituation of startle response under incremental sequence of stimulus intensities. J. Comp. Physiol. Psychol. 67, 486–492 5787400 10.1037/h0027308

[B17] Doucet J. R. Ryugo D. K. (1997). Projections from the ventral cochlear nucleus to the dorsal cochlear nucleus in rats. J. Comp. Neurol. 385, 245–264 9268126

[B18] Doucet J. R. Ryugo D. K. (2003). Axonal pathways to the lateral superior olive labeled with biotinylated dextran amine injections in the dorsal cochlear nucleus of rats. J. Comp. Neurol. 461, 452–465 10.1002/cne.10722 12746862

[B19] Dragunow M. Faull R. (1989). The use of c-fos as a metabolic marker in neuronal pathway tracing. J. Neurosci. Methods 29, 261–265 10.1016/0165-0270(89)90150-7 2507830

[B20] Fendt M. Li L. Yeomans J. S. (2001). Brain stem circuits mediating prepulse inhibition of the startle reflex. Psychopharmacology 156, 216–224 10.1007/s002130100794 11549224

[B21] Friauf E. Ostwald J. (1988). Divergent projections of physiologically characterized rat ventral cochlear nucleus neurons as shown by intraaxonal injection of horseradish peroxidase. Exp. Brain Res. 73, 263–284 10.1007/BF00248219 3215304

[B22] Furness D. N. Lawton D. M. (2003). Comparative distribution of glutamate transporters and receptors in relation to afferent innervation density in the mammalian cochlea. J. Neurosci. 23, 11296–11304 14672993 10.1523/JNEUROSCI.23-36-11296.2003 PMC6740530

[B23] Gómez-Nieto R. Horta-Junior J. A. Castellano O. Herrero-Turrión M. J. Rubio M. E. López D. E. (2008b). Neurochemistry of the afferents to the rat cochlear root nucleus: possible synaptic modulation of the acoustic startle. Neuroscience 154, 51–64 10.1016/j.neuroscience.2008.01.079 18384963 PMC2492054

[B24] Gómez-Nieto R. Horta-Júnior J. A. Castellano O. Sinex D. G. López D. E. (2010). Auditory prepulse inhibition of neuronal activity in the rat cochlear root nucleus, in The Neurophysiological Bases of Auditory Perception, eds López Poveda E. A. Palmer A. R. Meddis R. (New York, NY: Springer), 79–90 10.1007/978-1-4419-5686-6_8

[B25] Gómez-Nieto R. Rubio M. E. (2009). A bushy cell network in the rat ventral cochlear nucleus. J. Comp. Neurol. 516, 241–263 10.1002/cne.22139 19634178 PMC2841288

[B26] Gómez-Nieto R. Rubio M. E. López D. E. (2008a). Cholinergic input from the ventral nucleus of the trapezoid body to cochlear root neurons in rats. J. Comp. Neurol. 506, 452–468 10.1002/cne.21554 18041785

[B27] Gómez-Nieto R. Sinex D. G., C. Horta-Júnior J. D. Castellano O. Herrero-Turrión J. M. López D. E. (2013). A fast cholinergic modulation of the primary acoustic startle circuit in rats. Brain Struct. Funct. [Epub ahead of print]. 10.1007/s00429-013-0585-8 23733175

[B28] Gourevitch G. Hack H. M. (1966). Audibility in the rat. J. Comp. Physiol. Psychol. 62, 289–291 10.1037/h0023669 5969608

[B29] Gundersen H. J. Bendtsen T. F. Korbo L. Marcussen N. Møller A. Nielsen K. (1988). Some new, simple and efficient stereological methods and their use in pathological research and diagnosis. APMIS 96, 379–394 10.1111/j.1699-0463.1988.tb05320.x 3288247

[B30] Hackney C. M. Osen K. K. Ottersen O. P. Storm-Mathisen J. Manjaly G. (1996). Immunocytochemical evidence that glutamate is a neurotransmitter in the cochlear nerve: a quantitative study in the guineapig anteroventral cochlear nucleus. Eur. J. Neurosci 8, 79–91 10.1111/j.1460-9568.1996.tb01169.x 8713452

[B31] Harrison J. M. Warr W. B. Irving R. E. (1962). Second order neurons in the acoustic nerve. Science 138, 893–895 10.1126/science.138.3543.893 13952991

[B32] Hoffman H. S. Ison J. R. (1980). Reflex modification in the domain of startle: I. Some empirical findings and their implication for how the nervous system processes sensory input. Psychol. Rev. 87, 175–189 7375610

[B33] Horta-Júnior J. A. López D. E. Alvarez-Morujo A. J. Bittencourt J. C. (2008). Direct and indirect connections between cochlear root neurons and facial motor neurons: pathways underlying the acoustic pinna reflex in the albino rat. J. Comp. Neurol. 507, 1763–1779 10.1002/cne.21625 18260150

[B34] Ison J. R. McAdam D. W. Hammond G. R. (1973). Latency and amplitude changes in the acoustic startle reflex of the rat produced by variation in auditory prestimulation. Physiol. Behav. 10, 1035–1039 10.1016/0031-9384(73)90185-6 4717640

[B35] Kandler K. Herbert H. (1991). Auditory projections from the cochlear nucleus to pontine and mesencephalic reticular nuclei in the rat. Brain Res. 562, 230–242 10.1016/0006-8993(91)90626-7 1773339

[B36] Kelly J. B. Masterton B. (1977). Auditory sensitivity of the albino rat. J. Comp. Physiol. Psychol. 91, 930–936 10.1037/h0077356 893752

[B37] Koch M. (1999). The neurobiology of startle. Prog. Neurobiol. 59, 107–128 10.1016/S0301-0082(98)00098-7 10463792

[B38] Koch M. Schnitzler H. U. (1997). The acoustic startle response in rats–circuits mediating evocation, inhibition and potentiation. Behav. Brain Res. 89, 35–49 10.1016/S0166-4328(97)02296-1 9475613

[B39] Lang P. J. Bradley M. M. Cuthbert B. N. (1990). Emotion, attention, and the startle reflex. Psychol. Rev. 97, 377–395 2200076

[B40] Lee Y. López D. E. Meloni E. G. Davis M. (1996). A primary acoustic startle pathway: obligatory role of cochlear root neurons and the nucleus reticularis pontis caudalis. J. Neurosci. 16, 3775–3789 8642420 10.1523/JNEUROSCI.16-11-03775.1996 PMC6578836

[B41] Lehmann J. Pryce C. R. Feldon J. (1999). Sex differences in the acoustic startle response and prepulse inhibition in Wistar rats. Behav. Brain Res. 104, 113–117 10.1016/S0166-4328(99)00058-3 11125729

[B42] Leitner D. S. Cohen M. E. (1985). Role of the inferior colliculus in the inhibition of acoustic startle in the rat. Physiol. Behav. 34, 65–70 10.1016/0031-9384(85)90079-4 4034696

[B43] Li L. Frost B. J. (1996). Azimuthal sensitivity of rat pinna reflex: EMG recordings from cervicoauricular muscles. Hear. Res. 100, 192–200 10.1016/0378-5955(96)00119-0 8922994

[B44] Li L. Yue Q. (2002). Auditory gating processes and binaural inhibition in the inferior colliculus. Hear. Res. 168, 98–109 10.1016/S0378-5955(02)00356-8 12117513

[B45] Lingenhöhl K. Friauf E. (1994). Giant neurons in the rat reticular formation: a sensorimotor interface in the elementary acoustic startle circuit? J. Neurosci. 14, 1176–1194 8120618 10.1523/JNEUROSCI.14-03-01176.1994 PMC6577542

[B46] London M. Segev I. (2001). Synaptic scaling *in vitro* and *in vivo*. Nat. Neurosci. 4, 853–855 10.1038/nn0901-853 11528406

[B47] López D. E. Merchán M. A. Bajo V. M. Saldaña E. (1993). The cochlear root neurons in the rat, mouse and gerbil, in The Mammalian Cochlear Nuclei: Organization and Function, eds Merchán M. A. Juiz J. M. Godfrey D. A. Mugnaini E. (New York, NY: Plenum Press), 291–301

[B48] López D. E. Saldaña E. Nodal F. R. Merchán M. A. Warr W. B. (1999). Projections of cochlear root neurons, sentinels of the auditory pathway in the rat. J. Comp. Neurol. 415, 160–174 10545157

[B49] Malmierca M. S. Merchán M. Henkel C. K. Oliver D. L. (2002). Direct projections from the dorsal cochlear nucleus to the auditory thalamus in the rat. J. Neurosci. 22, 10891–10897 12486183 10.1523/JNEUROSCI.22-24-10891.2002 PMC6758437

[B50] Marsh R. Hoffman H. S. Stitt C. L. (1973). Temporal integration in the acoustic startle reflex of the rat. J. Comp. Physiol. Psychol. 82, 507–511 10.1037/h0034129 4706587

[B51] Meloni E. G. Davis M. (1998). The dorsal cochlear nucleus contributes to a high intensity component of the acoustic startle reflex in rats. Hear. Res. 119, 69–80 9641320 10.1016/s0378-5955(98)00040-9

[B52] Merchán M. A. Collía F. López D. E. Saldaña E. (1988). Morphology of cochlear root neurons in the rat. J. Neurocytol. 17, 711–725 2463341 10.1007/BF01260998

[B53] Murphy C. A. Feldon J. (2001). Interactions between environmental stimulation and antipsychotic drug effects on forebrain c-fos activation. Neuroscience 104, 717–730 10.1016/S0306-4522(01)00110-5 11440804

[B54] Nodal F. R. López D. E. (2003). Direct input from cochlear root neurons to pontine reticulospinal neurons in albino rat. J. Comp. Neurol. 460, 80–93 10.1002/cne.10656 12687698

[B55] Oertel D. Shatadal S. Cao X. J. (2008). In the ventral cochlear nucleus Kv1.1 and subunits of HCN1 are colocalized at surfaces of neurons that have low-voltage-activated and hyperpolarization-activated conductances. Neuroscience. 154, 77–86 10.1016/j.neuroscience.2008.01.085 18424000 PMC2493296

[B56] Oliver D. L. Ostapoff E. M. Beckius G. E. (1999). Direct innervation of identified tectothalamic neurons in the inferior colliculus by axons from the cochlear nucleus. Neuroscience. 93, 643–658 10.1016/S0306-4522(99)00143-8 10465448

[B57] Oliver D. L. Shneiderman A. (1991). The anatomy of the inferior colliculus, A cellular basis for integration of monaural and binaural information, in Neurobiology of Hearing, eds Altschuler R. A. Bobbin R. P. Clopton B. M. Hoffmann D. W. (New York, NY: Raven Press), 195–222

[B58] Osen K. K. López D. E. Slyngstad T. A. Ottersen O. P. Storm-Mathisen J. (1991). GABA-like and glycine-like immunoreactivities of the cochlear root nucleus in rat. J. Neurocytol. 20, 17–25 10.1007/BF01187131 1709203

[B59] Paxinos G. Watson C. (2009). The Rat Brain in Stereotaxic Coordinates, Compact 6th Edn. San Diego, CA: Elsevier Academic Press

[B60] Pirone A. Kart S. Zuccotti A. Rüttiger L. Pilz P. Brown D. H. (2014). α2δ3 is essential for normal structure and function of auditory nerve synapses and is a novel candidate for auditory processing disorders. J. Neurosci. 34, 434–445 10.1523/JNEUROSCI.3085-13.2014 24403143 PMC6608152

[B61] Popescu M. V. Polley D. B. (2010). Monaural deprivation disrupts development of binaural selectivity in auditory midbrain and cortex. Neuron 65, 718–731 10.1016/j.neuron.2010.02.019 20223206 PMC2849994

[B62] Potash M. Kelly J. (1980). Development of directional responses to sounds in the rat (*Rattus norvegicus*). J. Comp. Physiol. Psychol. 94, 864–877 7430470 10.1037/h0077819

[B63] Prosser C. L. Hunter W. S. (1936). The extinction of startle resnonses and spinal reflexes in the white rat. Am. J. Physiol. 117, 609–618

[B64] Rajakumar N. Elisevich K. Flumerfelt B. A. (1993). Biotinylated dextran: a versatile anterograde and retrograde neuronal tracer. Brain Res. 607, 47–53 10.1016/0006-8993(93)91488-E 7683244

[B65] Rubio M. E. (2006). Redistribution of synaptic AMPA receptors at glutamatergic synapses in the dorsal cochlear nucleus as an early response to cochlear ablation in rats. Hear. Res. 216–217, 154–167 10.1016/j.heares.2006.03.007 16644159

[B66] Rubio M. E. Juiz J. M. (2004). Differential distribution of synaptic endings containing glutamate, glycine, and GABA in the rat dorsal cochlear nucleus. J. Comp. Neurol. 477, 253–272 10.1002/cne.20248 15305363

[B67] Sagar S. M. Sharp F. R. Curran T. (1988). Expression of c-fos protein in brain: metabolic mapping at the cellular level. Science 240, 1328–1331 10.1126/science.3131879 3131879

[B68] Saint Marie R. L. Luo L. Ryan A. F. (1999). Effects of stimulus frequency and intensity on c-fos mRNA expression in the adult rat auditory brainstem. J. Comp. Neurol. 404, 258–270 9934998 10.1002/(sici)1096-9861(19990208)404:2<258::aid-cne9>3.0.co;2-u

[B69] Saul S. M. Brzezinski J. A. 4th. Altschuler R. A. Shore S. E. Rudolph D. D. Kabara L. L. (2008). Math5 expression and function in the central auditory system. Mol. Cell. Neurosci. 37, 153–169 10.1016/j.mcn.2007.09.006 17977745 PMC2266824

[B70] Sinex D. G. López D. E. Warr W. B. (2001). Electrophysiological responses of cochlear root neurons. Hear. Res. 158, 28–38 10.1016/S0378-5955(01)00293-3 11506934

[B71] Spruston N. (2000). Distant synapses raise their voices. Nat. Neurosci. 8, 849–851 10.1038/78734 10966607

[B72] Swerdlow N. R. Geyer M. A. Braff D. L. (2001). Neural circuit regulation of prepulse inhibition of startle in the rat: current knowledge and future challenges. Psychopharmacology 156, 194–215 10.1007/s002130100799 11549223

[B73] Szabo I. (1964). Analysis of the muscular action potentials accompanying the acoustic startle reaction. Acta Physiol. Hung. 27, 167–178

[B74] Wagner T. Pilz P. K. Fendt M. (2000). The superior olivary complex is necessary for the full expression of the acoustic but not tactile startle response in rats. Behav. Brain Res. 108, 181–188 10.1016/S0166-4328(99)00146-1 10701661

[B75] Walberg F. (1974). Crossed reticulo-reticular projections in the medulla, pons and mesencephalon. An autoradiographic study in the cat. Kidney Int. 5, 127–134 4606397 10.1007/BF00525765

[B76] Wang H. Yin G. Rogers K. Miralles C. de Blas A. L. Rubio M. E. (2011). Monaural conductive hearing loss alters the expression of the GluA3 AMPA and glycine receptor alpha1 subunits in bushy and fusiform cells of the cochlear nucleus. Neuroscience 199, 438–451 10.1016/j.neuroscience.2011.10.021 22044924 PMC3237936

[B77] Whiting B. Moiseff A. Rubio M. E. (2009). Cochlear nucleus neurons redistribute synaptic AMPA and glycine receptors in response to monaural conductive hearing loss. Neuroscience 163, 1264–1276 10.1016/j.neuroscience.2009.07.049 19646510 PMC2760652

[B78] Wilson N. R. Kang J. Hueste E. V. Leung T. Varoqui H. Murnick J. G. (2005). Presynaptic regulation of quantal size by the vesicular glutamate transporter VGLUT1. J. Neurosci. 25, 6221–6234 10.1523/JNEUROSCI.3003-04.2005 15987952 PMC6725055

[B79] Yeomans J. S. Frankland P. W. (1996). The acoustic startle reflex: neurons and connections. Brain Res. Rev. 21, 301–314 10.1016/0165-0173(96)00004-5 8806018

[B80] Yeomans J. S. Lee J. Yeomans M. H. Steidl S. Li L. (2006). Midbrain pathways for prepulse inhibition and startle activation in rat. Neuroscience 142, 921–929 10.1016/j.neuroscience.2006.06.025 16996220

[B81] Yeomans J. S. Li L. Scout B. W. Frankland P. W. (2002). Tactile, acoustic and vestibular systems sum to elicit the startle reflex. Neurosci. Biobehav. Rev. 26, 1–11 10.1016/S0149-7634(01)00057-4 11835980

[B82] Zhou J. Nannapaneni N. Shore S. (2007). Vesicular glutamate transporters 1 and 2 are differentially associated with auditory nerve and spinal trigeminal inputs to the cochlear nucleus. J. Comp. Neurol. 500, 777–787 10.1002/cne.21208 17154258

