# Supplementary figures and images for: Origin and function of short-latency inputs to the neural substrates underlying the acoustic startle reflex
Source: Front Neurosci. 2014 Jul 25;8:216. doi: 10.3389/fnins.2014.00216 (PMC4110630; doi:10.3389/fnins.2014.00216)

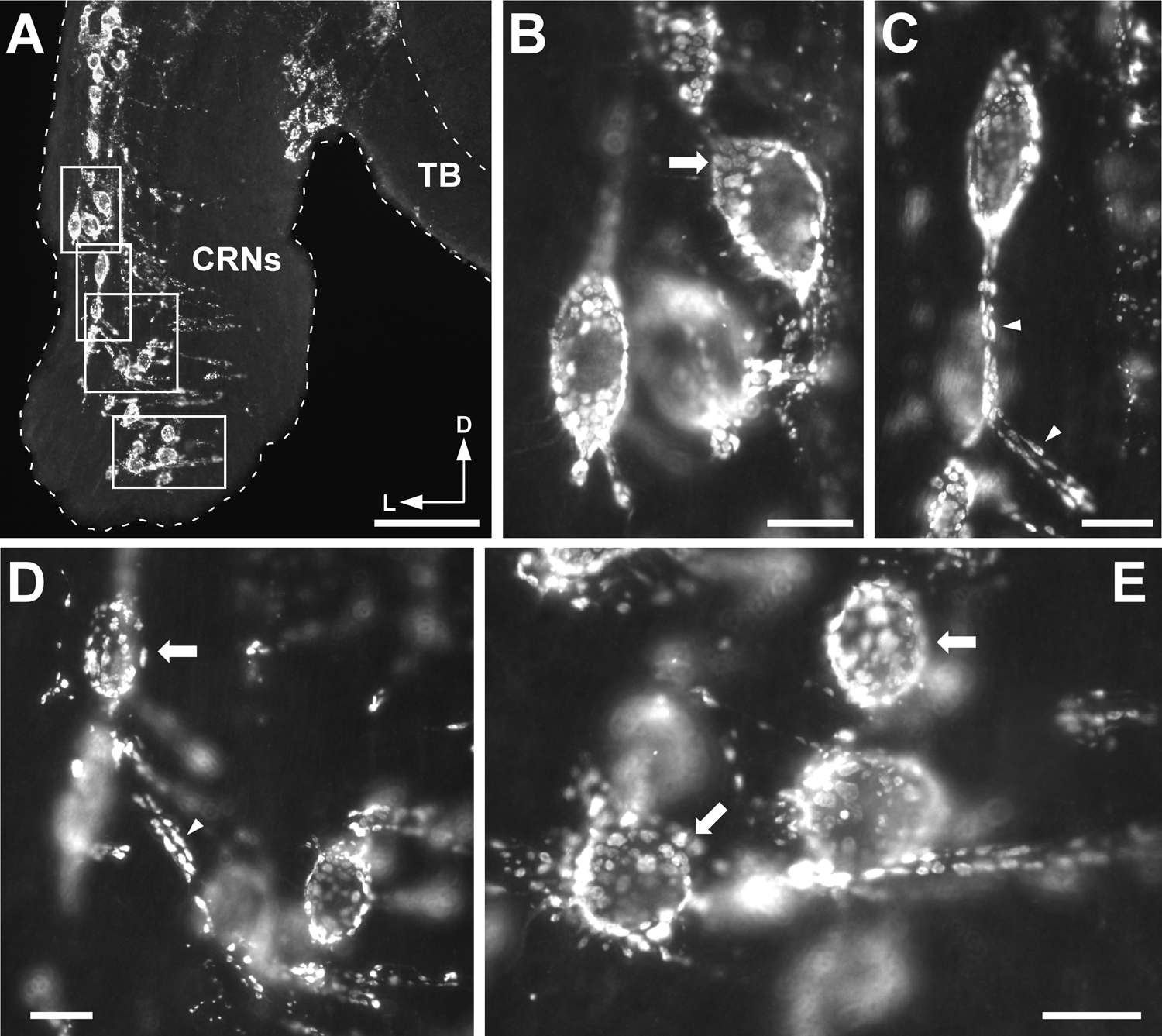

Supplement: Supplemental Figure 1 — Distribution of VGLUT1-immunolabeled endings in the cochlear root nucleus. (A) Epi-fluorescence micrograph of a coronal section shows VGLUT1-immunolabeled endings (Cy3 fluorochrome) in the cochlear root nucleus. (B–E) Epi-fluorescence micrographs of the boxed areas in (A) show distribution of VGLUT1-immunolabeled endings from dorsal to ventral regions of the cochlear root nucleus. Note that numerous VGLUT1-immunolabeled endings decorate unlabeled cell bodies (arrows) and dendrites (arrowheads) of cochlear root neurons (CRNs). TB, trapezoid body. Scale bars = 200μm in A; 25μm in B–E. [file DataSheet1.ZIP › Supplementary Figures/Figure S1.TIF]

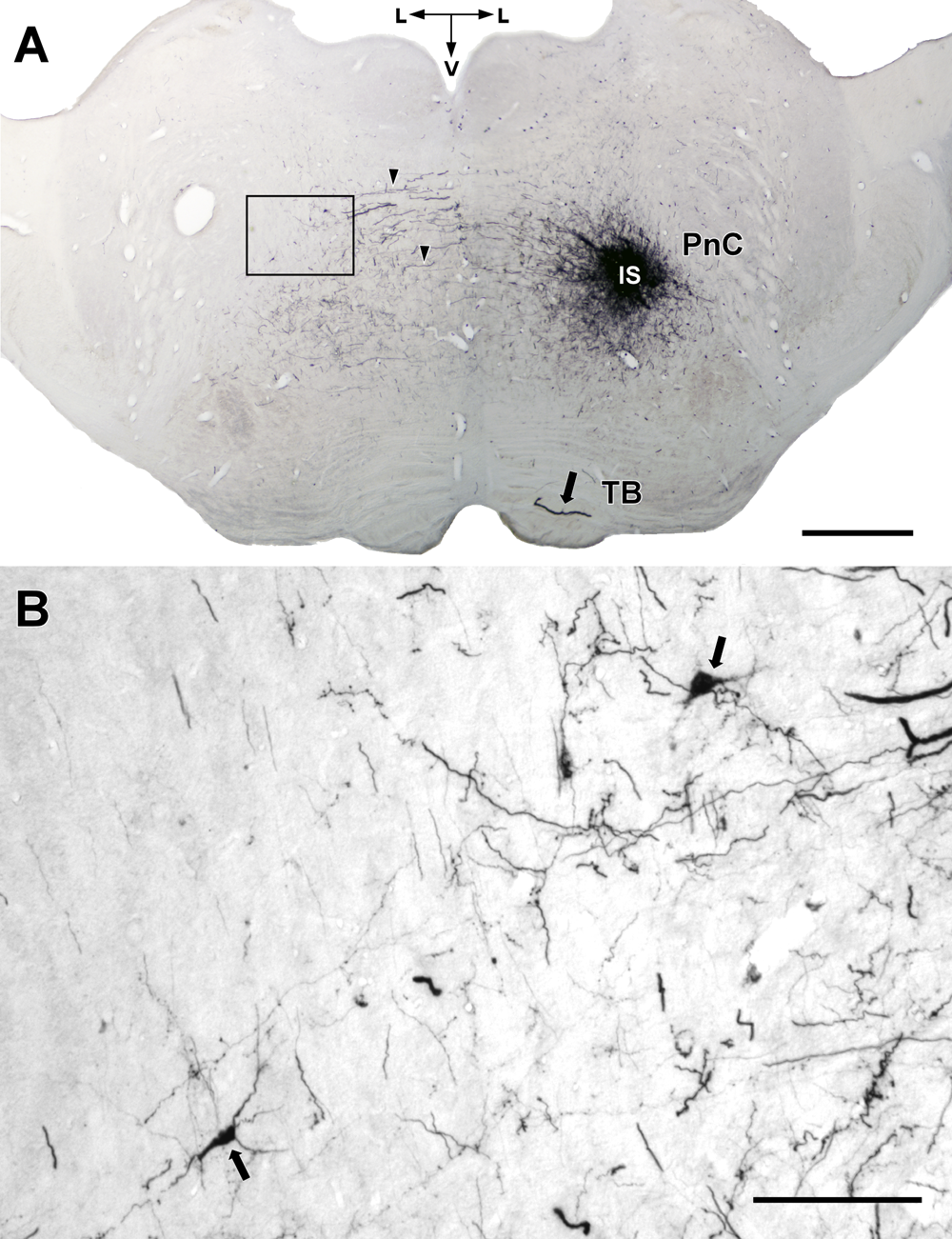

Supplement: Supplemental Figure 1 — Distribution of VGLUT1-immunolabeled endings in the cochlear root nucleus. (A) Epi-fluorescence micrograph of a coronal section shows VGLUT1-immunolabeled endings (Cy3 fluorochrome) in the cochlear root nucleus. (B–E) Epi-fluorescence micrographs of the boxed areas in (A) show distribution of VGLUT1-immunolabeled endings from dorsal to ventral regions of the cochlear root nucleus. Note that numerous VGLUT1-immunolabeled endings decorate unlabeled cell bodies (arrows) and dendrites (arrowheads) of cochlear root neurons (CRNs). TB, trapezoid body. Scale bars = 200μm in A; 25μm in B–E. [file DataSheet1.ZIP › Supplementary Figures/Figure S2.TIF]

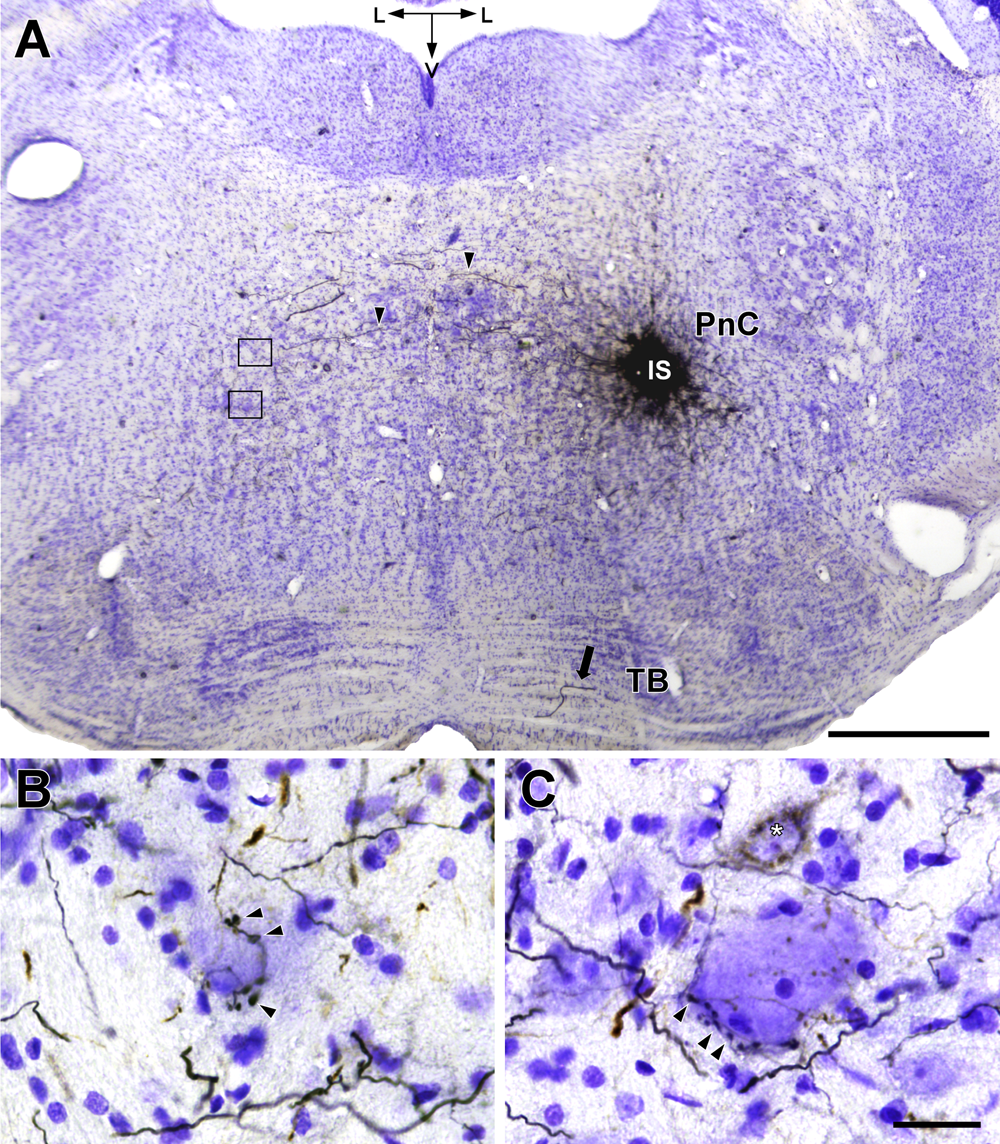

Supplement: Supplemental Figure 1 — Distribution of VGLUT1-immunolabeled endings in the cochlear root nucleus. (A) Epi-fluorescence micrograph of a coronal section shows VGLUT1-immunolabeled endings (Cy3 fluorochrome) in the cochlear root nucleus. (B–E) Epi-fluorescence micrographs of the boxed areas in (A) show distribution of VGLUT1-immunolabeled endings from dorsal to ventral regions of the cochlear root nucleus. Note that numerous VGLUT1-immunolabeled endings decorate unlabeled cell bodies (arrows) and dendrites (arrowheads) of cochlear root neurons (CRNs). TB, trapezoid body. Scale bars = 200μm in A; 25μm in B–E. [file DataSheet1.ZIP › Supplementary Figures/Figure S3.TIF]

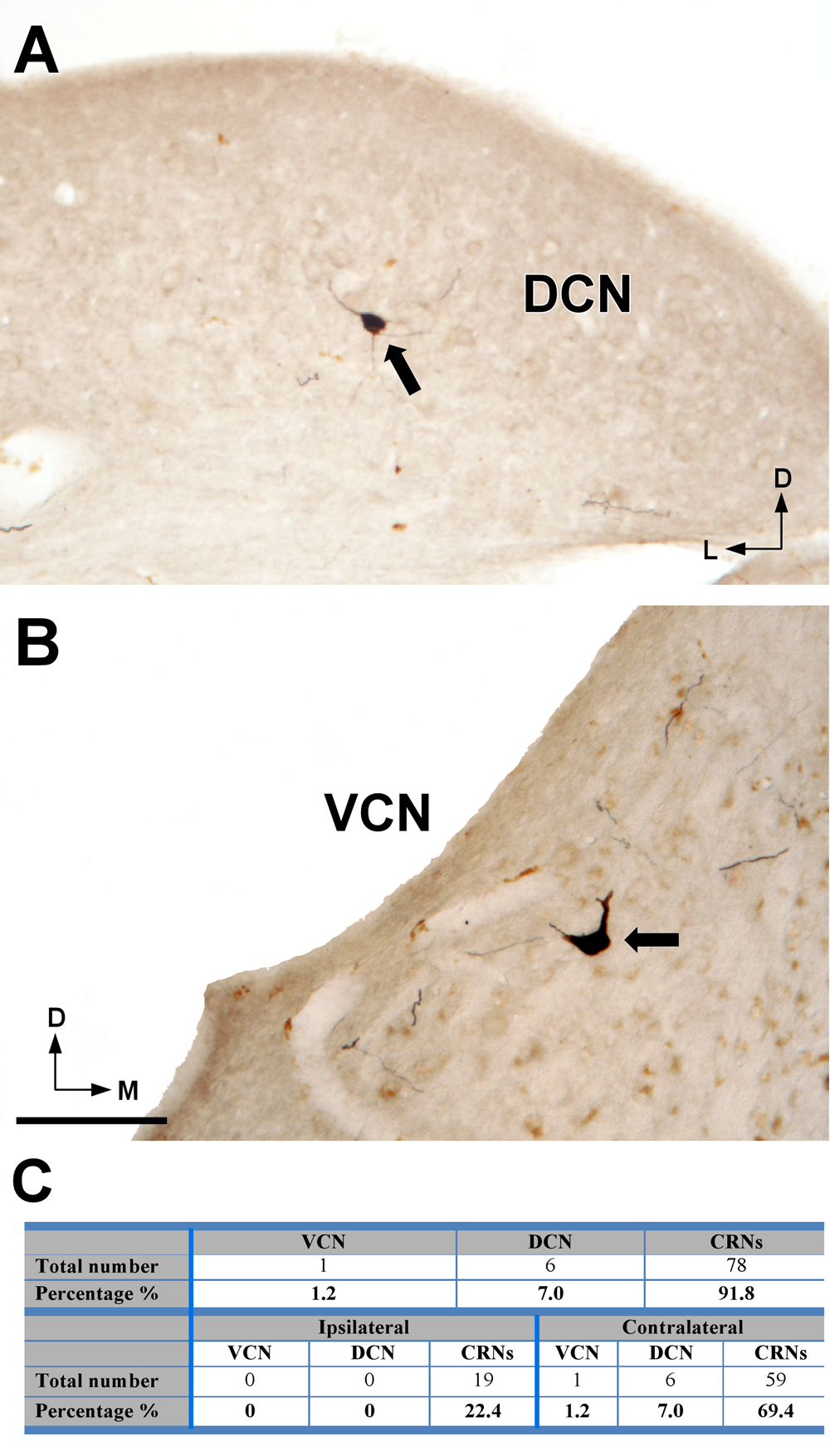

Supplement: Supplemental Figure 1 — Distribution of VGLUT1-immunolabeled endings in the cochlear root nucleus. (A) Epi-fluorescence micrograph of a coronal section shows VGLUT1-immunolabeled endings (Cy3 fluorochrome) in the cochlear root nucleus. (B–E) Epi-fluorescence micrographs of the boxed areas in (A) show distribution of VGLUT1-immunolabeled endings from dorsal to ventral regions of the cochlear root nucleus. Note that numerous VGLUT1-immunolabeled endings decorate unlabeled cell bodies (arrows) and dendrites (arrowheads) of cochlear root neurons (CRNs). TB, trapezoid body. Scale bars = 200μm in A; 25μm in B–E. [file DataSheet1.ZIP › Supplementary Figures/Figure S4.TIF]

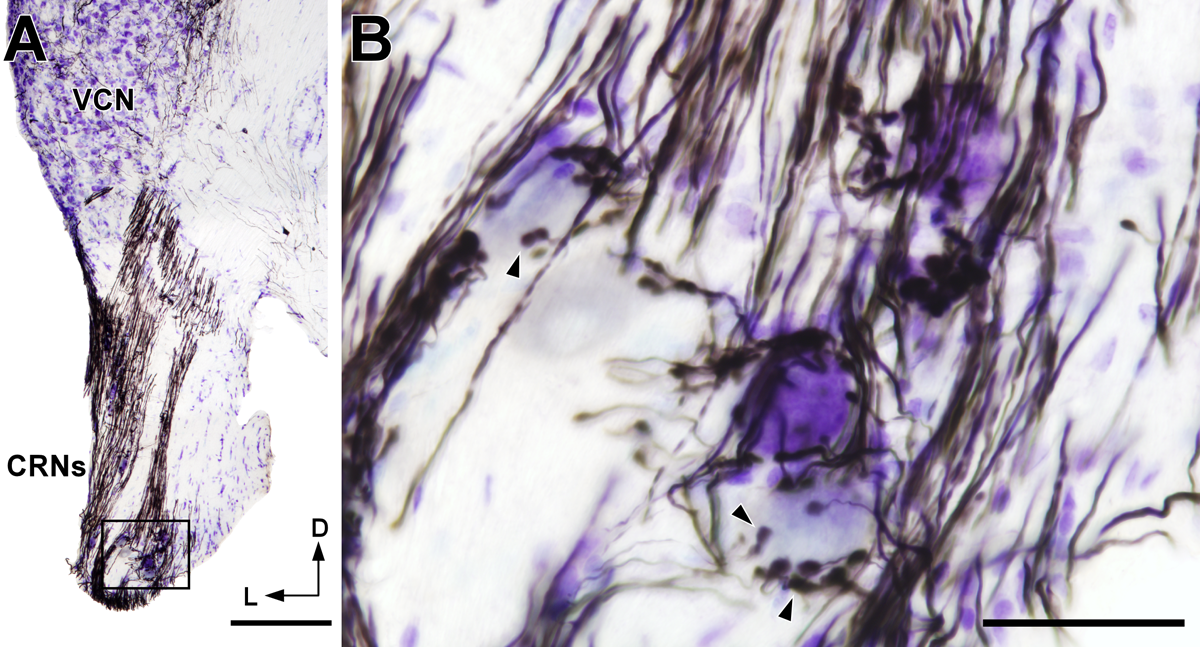

Supplement: Supplemental Figure 1 — Distribution of VGLUT1-immunolabeled endings in the cochlear root nucleus. (A) Epi-fluorescence micrograph of a coronal section shows VGLUT1-immunolabeled endings (Cy3 fluorochrome) in the cochlear root nucleus. (B–E) Epi-fluorescence micrographs of the boxed areas in (A) show distribution of VGLUT1-immunolabeled endings from dorsal to ventral regions of the cochlear root nucleus. Note that numerous VGLUT1-immunolabeled endings decorate unlabeled cell bodies (arrows) and dendrites (arrowheads) of cochlear root neurons (CRNs). TB, trapezoid body. Scale bars = 200μm in A; 25μm in B–E. [file DataSheet1.ZIP › Supplementary Figures/Figure S5.TIF]

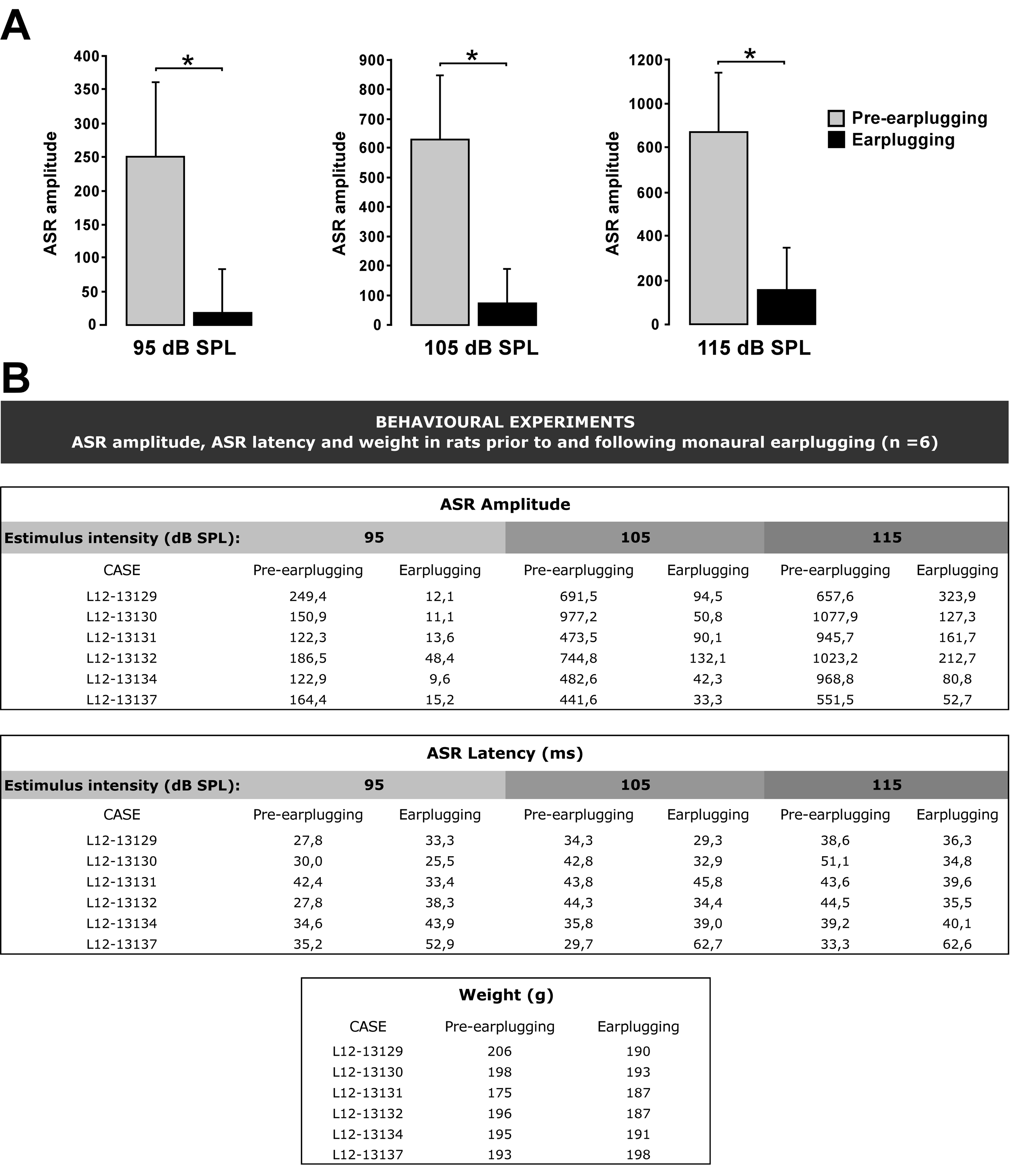

Supplement: Supplemental Figure 1 — Distribution of VGLUT1-immunolabeled endings in the cochlear root nucleus. (A) Epi-fluorescence micrograph of a coronal section shows VGLUT1-immunolabeled endings (Cy3 fluorochrome) in the cochlear root nucleus. (B–E) Epi-fluorescence micrographs of the boxed areas in (A) show distribution of VGLUT1-immunolabeled endings from dorsal to ventral regions of the cochlear root nucleus. Note that numerous VGLUT1-immunolabeled endings decorate unlabeled cell bodies (arrows) and dendrites (arrowheads) of cochlear root neurons (CRNs). TB, trapezoid body. Scale bars = 200μm in A; 25μm in B–E. [file DataSheet1.ZIP › Supplementary Figures/Figure S6.TIF]
